# Mapping the sociodemographic distribution and self-reported justifications for non-compliance with COVID-19 guidelines in the United Kingdom

**DOI:** 10.3389/fpsyg.2023.1183789

**Published:** 2023-07-19

**Authors:** Maria Bălăeț, Danielle L. Kurtin, Dragos C. Gruia, Annalaura Lerede, Darije Custovic, William Trender, Amy E. Jolly, Peter J. Hellyer, Adam Hampshire

**Affiliations:** ^1^Department of Brain Sciences, Imperial College London, London, United Kingdom; ^2^Neuromodulation Lab, Department of Psychology, Faculty of Health and Medical Sciences, University of Surrey, Guildford, United Kingdom; ^3^UKRI Centre for Doctoral Training in AI for Healthcare, Department of Computing, Imperial College London, London, United Kingdom; ^4^UK Dementia Research Institute: Care Research & Technology, London, United Kingdom; ^5^Engineering and Physical Sciences Research Council CDT Neurotechnology, Imperial College London, London, United Kingdom; ^6^NMR Unit, Queen Square Multiple Sclerosis Centre, UCL, Queen Square Institute of Neurology, Department of Neuroinflammation, Faculty of Brain Sciences, University College London, London, United Kingdom; ^7^Centre for Neuroimaging Sciences, Institute of Psychiatry, Psychology and Neuroscience, King’s College London, London, United Kingdom

**Keywords:** COVID-19, compliance, topic modelling, natural language processing, behaviour

## Abstract

Which population factors have predisposed people to disregard government safety guidelines during the COVID-19 pandemic and what justifications do they give for this non-compliance? To address these questions, we analyse fixed-choice and free-text responses to survey questions about compliance and government handling of the pandemic, collected from tens of thousands of members of the UK public at three 6-monthly timepoints. We report that sceptical opinions about the government and mainstream-media narrative, especially as pertaining to justification for guidelines, significantly predict non-compliance. However, free text topic modelling shows that such opinions are diverse, spanning from scepticism about government competence and self-interest to full-blown conspiracy theories, and covary in prevalence with sociodemographic variables. These results indicate that attempts to counter non-compliance through argument should account for this diversity in peoples’ underlying opinions, and inform conversations aimed at bridging the gap between the general public and bodies of authority accordingly.

## 1. Introduction

The COVID-19 pandemic has been characterised by a need for cooperation between citizens and bodies of authority in order to contain the virus and minimise health effects on the population. Achieving high compliance with the measures that governments put in place to mitigate infection and hospitalisation rates has proven to be a non-trivial challenge. For example, the United Kingdom rapidly achieved high rates of vaccination uptake, yet at the time of writing, a substantial minority still remain unvaccinated and at risk as COVID-19 infections are ongoing (Sonawane et al., 2021). Also, when in place, guidelines such as the mandate for wearing masks, social distancing in public spaces or using the NHS app were adhered to by many, but by no means all (Wright et al., 2022a). This raises the question of what reasons people commonly had for being non-compliant with guidelines and how such reasons varied across different segments of the general population.

A number of factors have been proposed to contribute to a reluctance to follow guidelines. These include perceptions of mixed messages from scientists, politicians and media regarding the optimal and appropriate level of control measures (Janssen and Van Der Voort, 2020), double standards in adherence to rules of government vs. general public, and corruption in the awarding of lucrative contracts. In parallel, social media platforms have acted as hubs for misinformation related to the pandemic, fuelling public unrest (Sharma et al., 2020) and a lack of compliance (Bridgman et al., 2020).

Indeed, on a general level, previous studies paint a bleak picture of public opinion on government and mainstream media handling of the pandemic, with research from British journalists highlighting polarised and often sceptical views (Nielsen et al., 2020). For example, an early survey of a representative sample of the United Kingdom’s population in April 2020 reported that 62% of respondents considered the government’s reaction to COVID-19 to be too slow, 31% did not trust the government to control the spread of COVID-19, and 24% did not believe the government had been truthful about COVID-19 (Duffy and Allington 2020). Some of the more extreme opinions go beyond perceptions of incompetence and self-interest to conspiracy theories of orchestrated and nefarious intent underpinning governmental response to the virus, the virus’ origins, or even its existence (Imhoff and Lamberty, 2020). This spectrum of sceptical opinions was likely to motivate non-compliant behaviours, with one study showing that distrust in government and higher levels of conspiratorial beliefs were associated with an unwillingness to engage in health protective behaviours and a reluctance to vaccinate against the virus (Murphy et al., 2021; Freeman et al., 2022; Gozgor, 2022; Han et al., 2023).

It remains unclear though, how the prevalence of non-compliant behaviours varied across different segments of society or evolved across time. Studies have associated different aspects of compliance with population variables including personality traits (Zajenkowski et al., 2020), political orientations (Goldstein and Wiedemann, 2020), and demographics (Clark et al., 2020; Enria et al., 2021; Wright et al., 2022b). However, other variables such as indicators of mental health, neuropsychiatric status, drug use and lifestyle factors also are likely relevant to compliance because they are known to shape peoples’ worldviews (Edmondson et al., 2011).

More critically, the most common opinions that motivated specific aspects of non-compliant behaviour are yet to be identified. Some past studies have applied traditional survey methods (Douglas et al., 2017; Sutton and Douglas, 2020, 2022), in which responses were given by selecting from pre-specified answers to pre-formulated questionnaire scales; for example, reflecting how strongly a respondent agrees with a particular statement. However, while fixed-answer approaches are convenient and appropriate in some contexts – e.g., when determining rates of compliance to specific measures – they have major limitations when seeking to ascertain the underlying opinions that motivate individuals to support or reject a given measure. This is because the answers that people can give are constrained to the options provided by the surveyor, which can bias participant responses towards a confirmation of surveyor views or provide little scope for communicating the most prevalent opinions (Butter and Knight, 2015).

A more computationally sophisticated approach that eschews researcher bias is to apply topic modelling to analyse free text data that are scraped from social media accounts, e.g., distilling in an unconstrained manner different prevalent opinions about the pandemic and its handling by bodies of authority in the form of “topics” (Cheng et al., 2021; Kwon and Park, 2022). This approach has provided novel insights into how misinformation propagates and fuels non-compliance (Cinelli et al., 2020; Janssen and Van Der Voort, 2020). However, as the free-text collected from social media is not written in response to targeted questions, the scope of derived topics can be quite broad, making it non-trivial to organise them within prominent pandemic themes, e.g., as different opinions on government guidelines or virus origins. Furthermore, social media data are prone to substantial biases; i.e., spamming, media and bots deliberately amplify specific views. Moreover, information for plotting how the prevalence of opinions varies with demographic and lifestyle variables is unreliable because people often mask aspects of their public-facing online identities (Tsao et al., 2021).

The aim of this study was to apply a novel approach that combines the strengths of both traditional survey and contemporary free text methods within the context of a large citizen science cohort in order to understand how sceptical opinions covary with specific aspects of non-compliant behaviour within the United Kingdom population (Hampshire et al., 2021a). First, we quantified the prevalence of sceptical statements endorsements as well as the prevalence of non-compliance with containment guidelines at different timepoints. Then, we used the fixed answer questions to produce scores for levels of non-compliance with government guidelines and scepticism about the mainstream government and media narrative on key pandemic themes and assess the strength of the relationship between them. We then employed a finer grain approach and show that different opinions pertaining to broad themes of scepticism have different predictive strength of non-compliance. To free text justifications underlying those statements we then applied a Latent Dirichlet Allocation algorithm, one of the most established methods for free text analysis (Blei et al., 2003), to distil the reasons people provided for being sceptical with respect to specific pandemic themes into the most commonly occurring topics. Finally, we quantified the degree to which the prevalence of these thematically-related topics evolved across three pandemic timepoints and covaried with sociodemographic variables.

## 2. Methodology

### 2.1. Study design

#### 2.1.1. Data collection and participant demographics

The Great British Intelligence Test commenced in December 2019 and recruited participants *via* direct advertisement on the BBC homepage and via exposure on a special BBC2 Horizon documentary (Hampshire et al., 2021c). The recruitment period lasted for 2 months and included two particular waves of recruitment – in December 2019 and in May 2020 (at a time when the United Kingdom was already 3 months into the COVID-19 pandemic). At those timepoints only sociodemographic information and brief lifestyle questions were presented to participants alongside the cognitive testing.

In December 2020 the questionnaires of ‘the Great British Intelligence Test’ were expanded to include additional variables related to key themes of interest identified during the early stages of the COVID-19 pandemic from early communications written on this topic (Nielsen et al., 2020). These pandemic-related questions were of three types: i) regarding people’s opinions about the pandemic handling by authorities, vaccines and the origins of COVID-19, ii) regarding people’s compliance with the measures suggested to reduce the spread of COVID-19, and iii) regarding people’s primary information source on the pandemic. The questions concerning people’s opinions triggered a free-text question in case people picked what was expected to be the “minoritarian” answer to allow them to explain the motives behind their choice.

Data was analysed from 20,922 individuals in December 2020, from 12,796 individuals in June 2021 and from 14,090 individuals in January 2022 who completed all questions that are subject of this study (Table 1). These represent the total of individuals who responded to our recontact at each of the timepoints of assessment part of the original cohort. Their demographics are shown in the Supplementary Table S1.

**Table 1:** Full study recruitment timeline and sample sizes.

		December 2019	May 2020	December 2020–January 2022
	Recruited	251,659	130,190	30,754
December 2020	Recontacted	95,441		N/A
	Responded	33,227		
	Complete responses	20,922		
June 2021	Recontacted	95,044		
	Responded	33,200		
	Complete responses	12,796		
January 2022	Recontacted	124,496		
	Responded	25,137		
	Complete responses	14,090		

In January 2020 the study was promoted by the BBC on the main and on the news homepages. Subsequently, in May 2020, the study was promoted again via the same channels and by AH on an episode of the BBC2 Horizon Documentary named “the Great British Intelligence Test.” Online links remained in prominent positions over the subsequent month in each promotion launch. The study survey consisted of a set of bespoke cognitive assessment tasks and of questionnaires including items related to socio-demographic and mental health variables. At the end of the survey, participants who wished to be recontacted for future studies were asked for their email addresses. Out of the total of the 765,830 participants recruited in December 2019 and May 2020 only 95,441 signed up with their email addresses and gave permission to be recontacted. The totality of the participants is detailed in Table 3. All 95,441 participants who signed up with their email addresses were recontacted in December 2020. 397 of those deleted their accounts between December 2020 and June 2021 when the next recontact happened. 1,303 further deleted their accounts between June 2021 and January 2022. In January 2022 people who signed up to the Great British Intelligence Test between December 2020 and January 2022 were also part of the recontacting scheme. At the recontacting in January 2022 104,516 people received a second email asking for their responses.

The survey was programmed in HTML5 with JavaScript and deployed *via* our Cognitron server system, which already supports diverse online studies. Cognitive and mental health results have been reported elsewhere (Hampshire, 2020; Chamberlain et al., 2021; Hampshire et al., 2021a,b; Bălăeț et al., 2023).

This study was run in accordance with the Helsinki Declaration of 1975, as revised in 2008. All procedures were approved by the Imperial College Research Ethics Committee (17IC4009). All participants provided informed consent prior to completing the survey.

#### 2.1.2. Survey

The pandemic-related questions relevant to the present work which were added to the Great British Intelligence Test in December 2020 are reported, alongside their scoring in the Supplementary Materials Part 4. The information sources question was dropped in January 2022.

### 2.2. Statistical methods

#### 2.2.1. Scoring for levels of sceptical opinions and non-compliance

We wanted to derive a composite score representative of the overall level of sceptical opinions and non-compliance of an individual. We scored the possible answers to the opinion and compliance questions on a continuous scale from 0 to 2, with 0 being fully compliant, depending on the question. Next, for each individual we evaluated the score for each question based on their answers and we added up all the scores to get a unique composite score. The sceptical opinion and non-compliance composite scores were, therefore, assigned on a scale from 0 to 4 and 0 to 10 respectively, where 0 means fully in agreement with the majority/compliant, 4 means having different opinions to the majority on all COVID-19 related matters and 10 means non-compliant at all with the government suggestions regarding conduit during the COVID-19 pandemic. The scoring system for each question can be found in Supplementary Materials Part 5.

#### 2.2.2. Predicting lack of compliance on the basis of sceptical opinion

To investigate whether any of the opinions as phrased by the fixed-answer opinion questions were predictive of non-compliance with the COVID-19 restrictions, we have fitted a linear regression model taking as predictors the endorsement of specific different sceptical opinions. We used the Python statmodels library (Georgiou et al., 2020).

#### 2.2.3. Topic modelling on free-text data

For the sceptical opinion and vaccine questions, in case of a particular response (in most cases what was expected to be the minoritarian response), respondents were asked to specify the rationale driving their answers. Topic modelling was used to exploit and extract the major classes of opinions justifying sceptical answers from the free-text data in an unsupervised fashion. For each question the following topic modelling pipeline was applied: a) pre-processing of the free-text data, b) selection of the optimal number of topics, c) implementation of LDA as topic modelling algorithm, d) rating of top 20 words and opinions per topic, and e) interpretation of top opinions to label/name the topic.

The following subsections cover this in more detail.

##### 2.3.3.1. Free-text data pre-processing

Free-text data was screened manually for non-related responses/spam responses and those entries were removed. Established natural language processing techniques were applied to pre-process the text data using the NLTK package in Python (Bird et al., 2009). These involved removing stop words, junk words, punctuation, special characters, numbers, words that occurred less than 10 times and lowercasing and lemmatizing the remaining text (bringing each word to its dictionary form). Data was then tokenized and vectorised (bag of words model).

##### 2.3.3.2. LDA implementation and selection of optimal number of topics

LDA falls under the rubric of topic modelling, a popular approach for both social network analysis and mapping out public opinions from free-text published on social media platforms (Albalawi et al., 2020).

LDA is the most popular algorithm for segregating free text data into topics; it uses a Dirichlet distribution prior to infer latent topics from a set of data (Blei et al., 2003). Broadly speaking, LDA analyses a *corpus* of *texts* (in our case, the collection of free-text responses to a given question) to identify *latent topics* – putatively, themes or topics which appear across multiple texts within the corpus (we interpret latent topics here as common justifications for rejecting commonly held opinions). Crucially, in contrast to fixed-answer questionnaires alone, this approach allows for important classes of public opinion to be identified *a posteriori*. LDA works on the assumption that documents input to the model are composed of some common underlying mixture of topics and seeks to both derive those latent topics and quantify each document’s relationship to them (Albalawi et al., 2020).

To group the opinions of the members of the British public into topics, we ran LDA on our data. Coherence measures identify the optimal number of topics in the dataset. We used the Cv coherence measure previously shown to be the most highly correlated with human judgement (Röder et al., 2015; Syed and Spruit, 2017) than other measures, such as algorithmic perplexity (Röder et al., 2015).

For finding latent topics in the free-text data we used the LDA multicore implementation in gensim (Rehurek and Sojka, 2010) which uses online LDA (Hoffman et al., 2010), an implementation based on online, stochastic, multithreaded optimisation with the intent of speeding up the computational inference process.

##### 2.3.3.3. Topic modelling evaluation

The free-text data was segmented into word pairs for the first step of computing Cv coherence. To calculate Cv coherence the probability that each word pair belongs to a certain topic was computed as well as a measure that quantifies how strongly a wordset supports another word set (Syed and Spruit, 2017).

##### 2.3.3.4. Topics labelling and opinions interpretation

The saved LDA models were used to infer the topic distribution in the opinions belonging to the newest time points namely June 2021 and January 2022. The topic mixture, or percentage that each opinion belongs to its assigned topic, was used to determine the 20 opinions that most strongly relate to each topic. These opinions were used as a corpus to perform thematic analysis, where topic labels were generated using thematic analysis as described in Hodge (1975). For each topic, the opinions were coded, and themes were extracted from the codes. Traditional thematic analysis often results in several themes generated from the corpus; due to the size of our corpus, we often extracted 1–2 themes. The most dominant theme was used as the label for each topic. Thematic analysis was independently performed by DK, DC, and AL, and the topic labels (themes) were compared to generate a consensus topic label.

## 3. Results

Participants were recruited in response to emails sent in December 2020, June 2021 and January 2022 to 95,441 participants who had signed up for the Great British Intelligence Test between December 2019–May 2020 (Hampshire, 2020). We analysed data from 20,922 respondents in December 2020, from 12,796 respondents in June 2021 and from 14,090 respondents in January 2022 using the Cognitron online assessment platform who total the number of participants who responded to our emails at each timepoint of assessment (Chamberlain et al., 2021; Hampshire et al., 2021a,b). Out of these individuals 2,797 respondents completed all three timepoints. Demographics of participants at all timepoints are presented in Supplementary Table S1.

### 3.1. Characterising distributions of sceptical opinions, non-compliant behaviours and information sources using fixed answer questions

**Figure 1:**
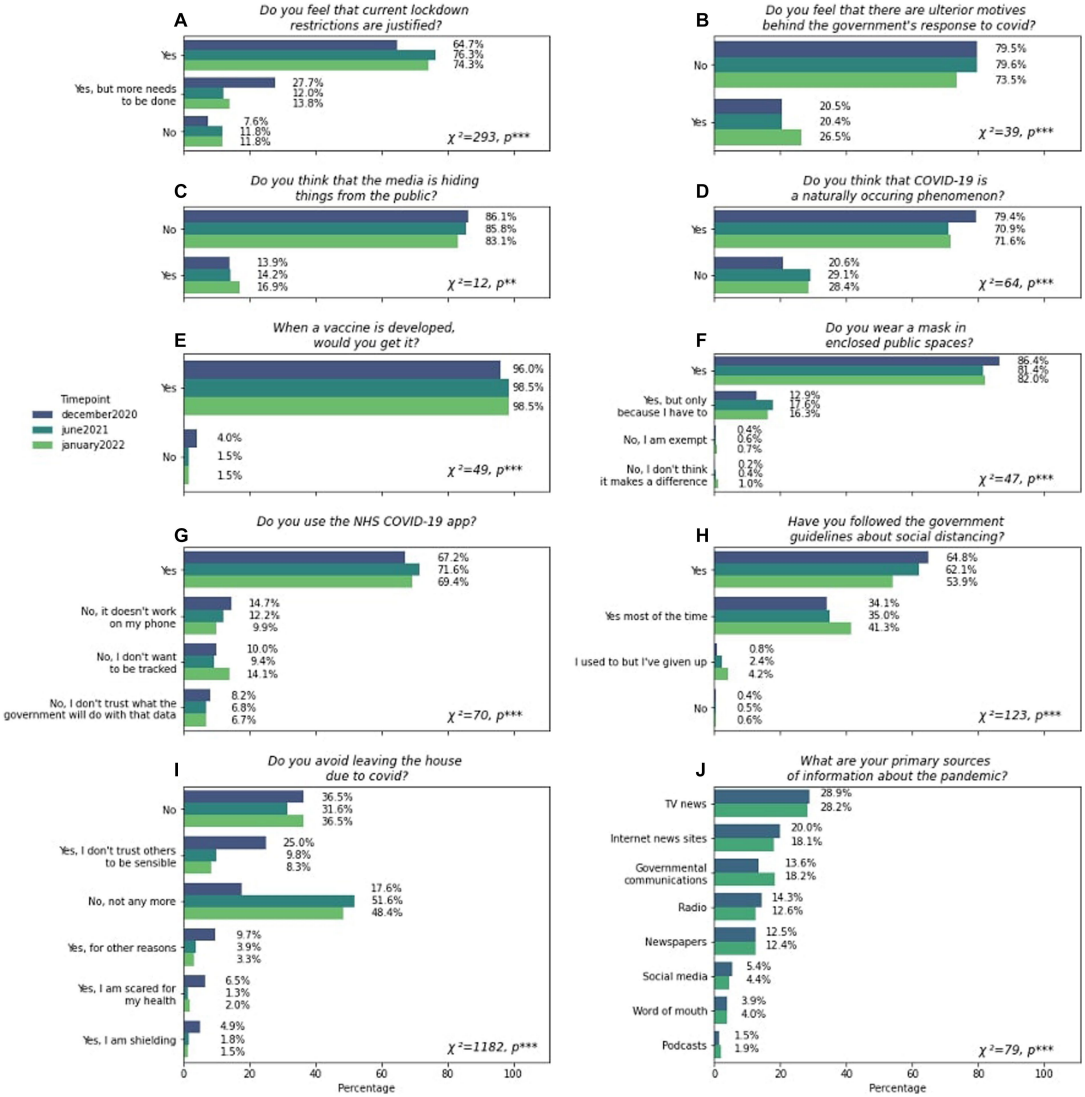
Distribution of scepticism, non-compliance and media sources. **(A–D)** illustrate the proportions of various responses to fixed-answer questions pertaining to scepticism about key pandemic related themes. **(E–I)** illustrate responses received to questions regarding compliance to measures that attempt to reduce COVID-19 transmission. **(J)** illustrates the distribution of primary sources about the pandemic that people use. All x-axes show percentages. Each panel contains χ^2^ and *p* values of tests for dissimilarity between the December 2020, June 2021 and January 2022 data. Answers are displayed in cascade order based on the most prevalent answers given in December 2020. Information regarding sources of information about the pandemic was not collected in January 2022. Significance is denoted as *, **, and *** for *p* < 0.05, *p* < 0.01, and *p* < 0.001, respectively.

We assessed the distribution of responses to three categories of fixed answer questions (Figure 1). The first category of question probed whether respondents endorsed ‘sceptical statements about the government and mainstream media narrative on broad pandemic themes. These were designed to probe whether respondents agreed that (A) the government actions in imposing lockdown restrictions either were justified or conversely were insufficient, (B) there were ulterior motives behind the government’s response to the pandemic, (C) the media were deliberately hiding things from the public, (D) the origin of SARS-CoV-2 was natural. The second category of question probed respondents compliance with specific measures to reduce virus transmission, specifically, (E) being vaccinated, (F) wearing masks, (G) using the NHS COVID-19 App, (H) following guidelines on social distancing, and (I) avoiding leaving the house. The third category comprised a single question probing respondents’ primary sources of information regarding the pandemic.

The majority of respondents indicated that they favoured government and mainstream-media intentions and actions during the pandemic (Figures 1A–D); however, there were substantial minorities who were more sceptical. For example, in December 2020, 7.6% agreed that the government imposed restrictions were not justified and 27.7% that their actions were insufficient (Figure 1A); 20.5% of respondents endorsed that there were ulterior motives behind the government’s actions in response to the pandemic (Figure 1B); 17% endorsed that the media were deliberately covering up information (Figure 1C); and 20.6% did not consider COVID-19 to be a natural phenomenon (Figure 1D). Over the course of 1 year there were statistically significant changes in the proportions of respondents to questions such as those about the ulterior motives behind governmental action (*χ*^2^ = 39, *p* < 0.001) and about COVID-19 not being a natural phenomenon (*χ*^2^ = 64, *p* < 0.001). Most notably, more respondents reported that the government had ulterior motives behind their response to the pandemic (the proportion rose from 20.5 to 26.5%) and that the COVID-19 pandemic had unnatural origins (the proportion rose from 20.6 to 28.4%). Conversely, fewer respondents considered that more needed to be done regarding restrictions on social behaviour in January 2022 (13.8%) compared to December 2020 (27.7%).

A similar pattern of results was evident for the fixed answer compliance questions which also saw significant changes in the proportion of respondents endorsing specific answers over time for questions pertaining to leaving the house (*χ*^2^ = 1,182, *p* < 0.001), using the Track and Trace app (*χ*^2^ = 70, *p* < 0.001), taking the vaccine (*χ*^2^ = 49, *p* < 0.001), or following social distancing guidelines (*χ*^2^ = 123, *p* < 0.001). Substantial minorities reported non-compliance with government measures to reduce virus transmission (Figures 1F–I). For example, in December 2020, 36.5% of people surveyed stated that they did not avoid leaving the house due to COVID-19 (Figure 1I), 10% of respondents did not want to be tracked with the NHS Track and Trace app, with 8.2% stating they did not trust what the government would do with that data (Figure 1G). However, only 4% of people stated that they would not get vaccinated when a vaccine became available (Figure 1E). Although only 0.4% of respondents reported not following social distancing guidelines at all, 34.1% reported not doing so all of the time (Figure 1H). Similarly, although 0.2% of responders reported not wearing a mask (Figure 1F) 12.9% only did so because they had to, a percentage that increased to 17.6% in June. Reflecting updates in government guidance, the most striking change from December 2020 to January 2022 was that people no longer avoided leaving their houses due to COVID-19, from 17.6 to 51.6% in June 2021 and 48.4% in January 2022 (Figure 1I).

In December 2020 the most common reported source of news about the pandemic was via TV (28.9%), and the Internet (20%), with social media accounting for 5.4% of responders’ primary source of information surrounding the pandemic (Figure 1J). These proportions changed minimally, although significantly (*χ*^2^ = 79, *p* < 0.001), across time. Specifically, marginally more respondents took their information about COVID-19 from governmental communications (from 13.6 to 18.2% from December 2020 to June 2021), podcasts (from 1.5 to 1.9% from December 2020 to June 2021) and word of mouth (from 3.9 to 4% from December 2020 to June 2021) and less from other sources such as TV news (from 28.9 to 28.2% from December 2020 to June 2021), internet news sites (from 20 to 18.1% from December 2020 to June 2021), radio (from 14.3 to 12.6% from December 2020 to June 2021), newspapers (from 12.5 to 12.4% from December 2020 to June 2021) or social media (from 5.4 to 4.4% from December 2020 to June 2021).

### 3.2. Predicting rates of non-compliance and levels of scepticism

**Figure 2:**
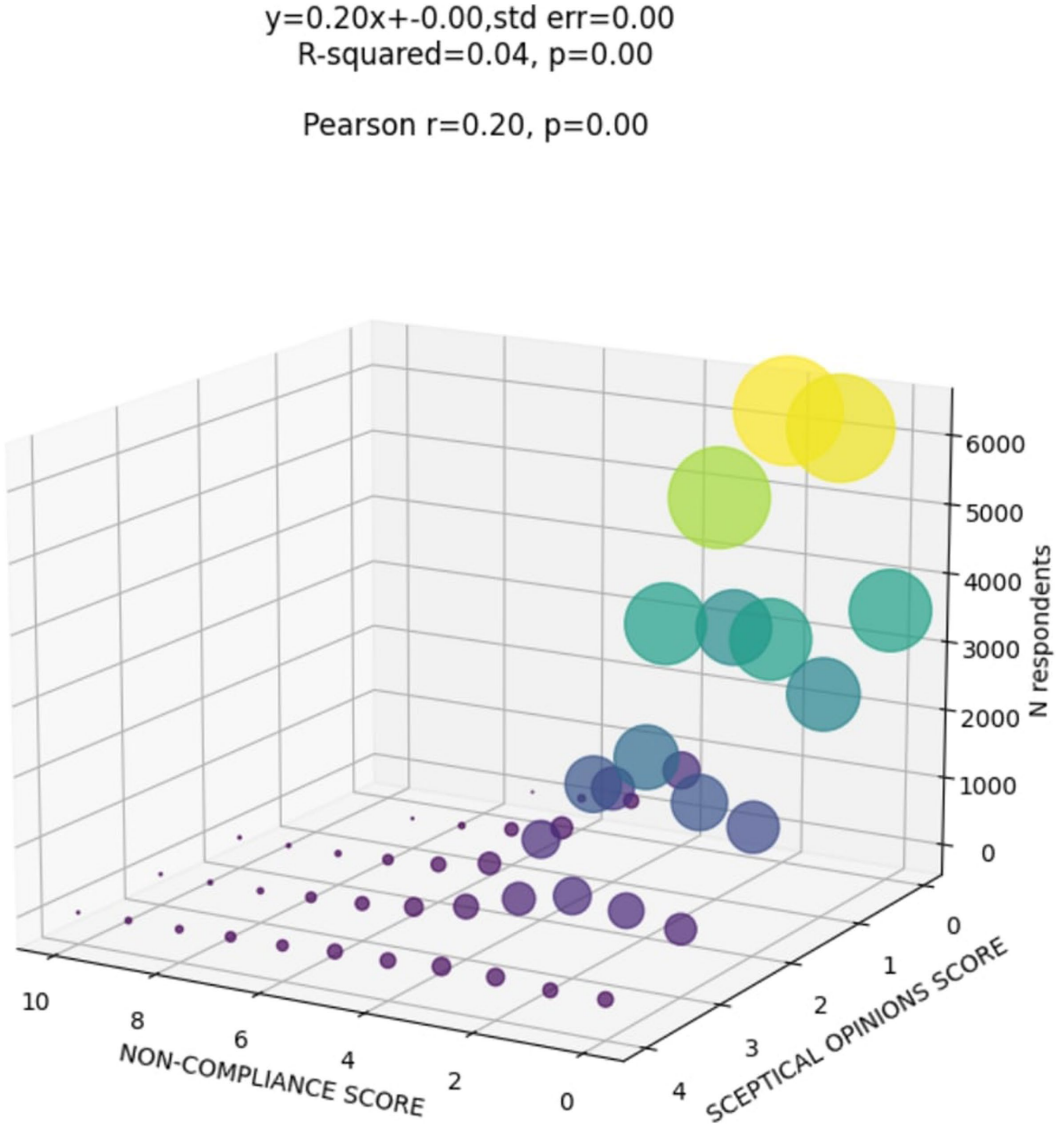
The relationship between scepticism and non compliance scores.

**Figure 3:**
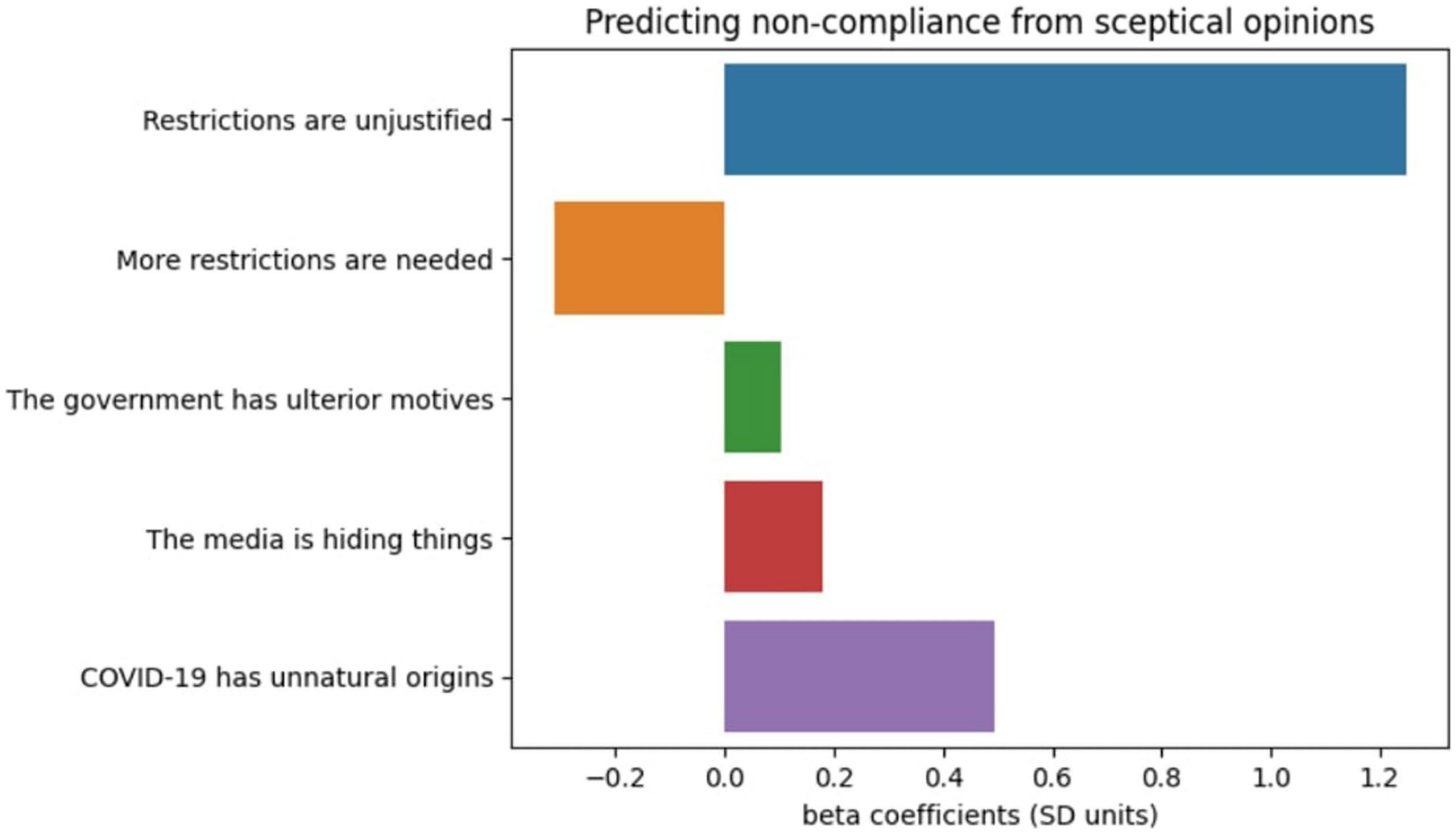
The relationship between specific sceptical statements endorsement and non-compliance score.

Next, we investigated whether differences in respondents’ non-compliance with COVID-19 safety measures could be predicted from i) their scepticism scores (Figure 2) or ii) endorsing sceptical statements about specific pandemic themes (restrictions, ulterior motives behind governmental action, media hiding things or the COVID-19 origins) (Figure 3).

#### 3.2.1. Predicting non-compliance on the basis of sceptical opinions

The answers given to the scepticism and non-compliance questions were aggregated into a composite score, adjusted for timepoint when the data were collected using a linear regression model and standardised (see Methods). The proportion of variation explained by timepoint was low for both scepticism (*R*^2^ = 0.01, *p* < 0.001) and non-compliance (*R*^2^ = 0.006, *p* < 0.001). Further, we identify that the relationship between the scepticism and noncompliance was weak but notable, characterised by a Pearson’s correlation coefficient of 0.2 (Figure 2).

#### 3.2.2. Predicting non-compliance on the basis of specific sceptical statements

Then, a multiple linear regression was fitted to predict this standardised non-compliance score with yes/no responses to each scepticism statement as predictors (*R*^2^ = 0.15, *p* < 0.001) (Figure 3).

There were statistically significant main effects (all *p* < 0.001) for each of these predictors. Specifically there was a significant effect of considering the restrictions unjustified (effect size 1.25SD, *F*_(1,47,797)_ = 4631.4, *p* < 0.001), considering more had to be done (effect size −0.31SD, F_(1,47,797)_ = 470.34, *p* < 0.001), thinking the government has ulterior motives (effect size 0.1SD, F_(1,47,797)_ = 47.67, *p* < 0.001), thinking the media is hiding things (effect size 0.18SD, F_(1,47,797)_ = 112.08, *p* < 0.001) or considering the origins of the virus unnatural (effect size 0.49SD, F_(1,47,797)_ = 1441.84, *p* < 0.001).

The greatest effect size was for endorsement of the statement that restrictions were unjustified, which according to Sawilowsky’s updated version of Cohen’s notion of effect sizes (Sawilowsky, 2009, 0.1 SD = very small, 0.2 SD = small, 0.5 SD = medium, 0.8 SD = large, 1.2 SD = very large and 2.0 SD = huge) was in the very large range at >1.2SDs. Conversely, the view that more restrictions were needed had the opposite relationship at ~ − 0.31SDs. Endorsement of the view that COVID-19 had unnatural origins had a small-medium effect size at ~0.4SDs. Endorsement of the views that government had ulterior motives and media were hiding things both had negligible scaled effect sizes.

Associations between sociodemographic predictors and scepticism or non-compliance are presented in Supplementary Table S6.

### 3.3. A topic-modelling analysis of free text responses

Topic modelling was applied to free-text responses to determine the most prevalent justifications that people gave in their own words for endorsing statements about the government having ulterior motives, the media hiding things and COVID-19 having unnatural origins or for being non-compliant with the vaccination guidelines. Specifically, if respondents indicated such views in their fixed answer responses, they were asked to justify those responses in their own words in a free text box. LDA models were trained separately on each of the resultant question-specific sets of free-text data. In this manner, the responders’ most common topics underlying sceptical opinions as pertained to the specific ‘theme’ question were distilled in the form of latent documents or “topics” in a data-driven manner. Preprocessing included removal of infrequent words, non-words, lemmatising and tokenization of text. The optimal number of topics for each question was defined by running the LDA model at all levels of complexity from one to thirty potential topic splits and identifying the one that produced the highest CV coherence value (a measure of co-occurrence of certain words that indicates what an ideal split of topics would be and correlates with human judgement) for a certain topic split (Syed and Spruit, 2017). Meaning of the topics was then inferred through thematic analysis (Braun and Clarke, 2006) performed independently by the authors of the top twenty opinions that aligned best with each latent topic (see Methods). Each individual free text response was categorised as belonging to the topic for which it had the highest loading within the corresponding LDA.

#### 3.3.1. Distribution of topics underlying sceptical opinions and refusal to Get vaccinated across timepoints

**Figure 4:**
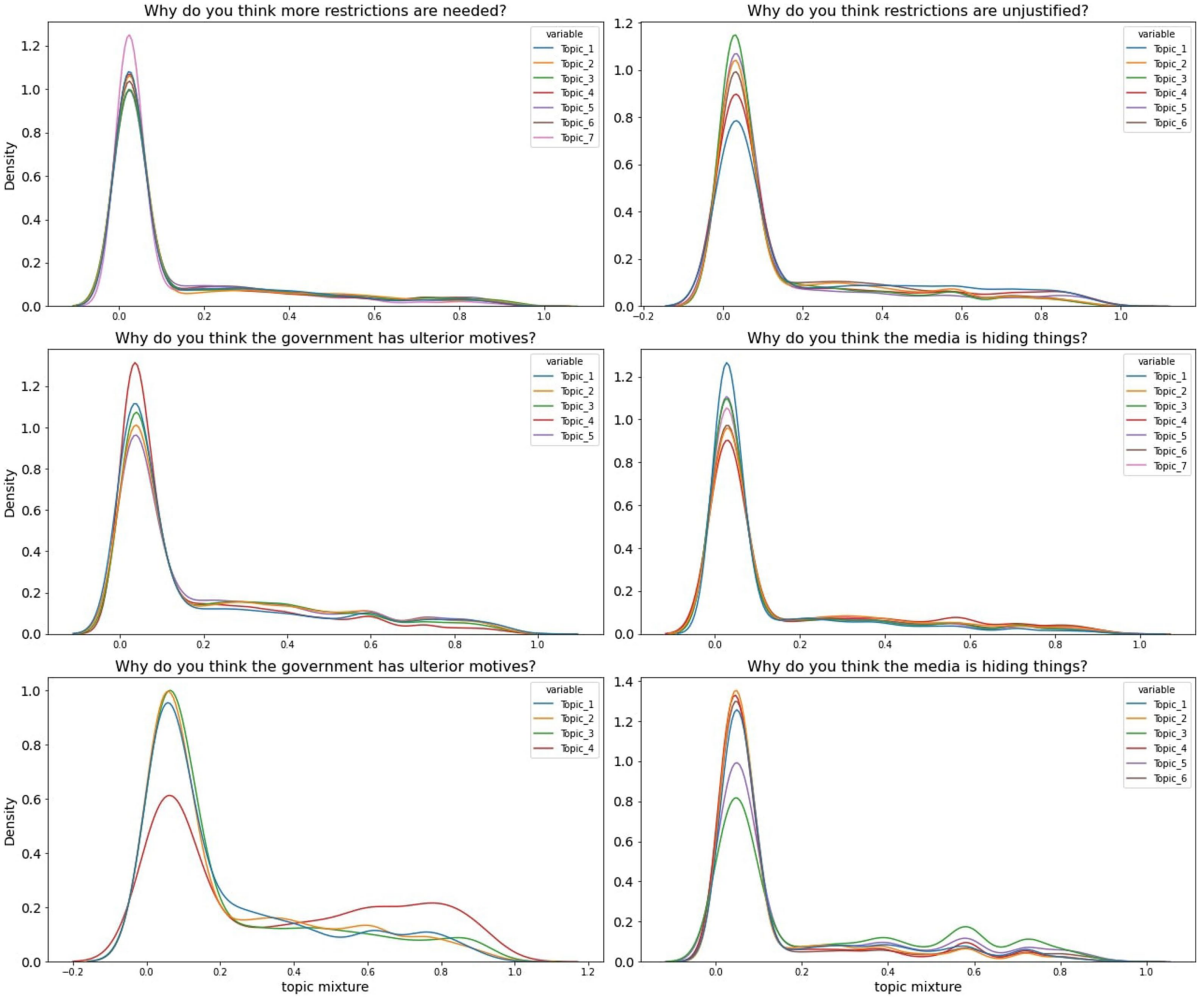
Topic probability LDA mixtures distribution. Topic labels correspond to topics described in Table 2.

The prevalence of the dominant LDA topics for each question and at each time point are illustrated in Table 2, whereas the distribution of topic mixtures is illustrated in Figure 4. Chi-squared tests confirmed that changes in the prevalence of topics were significant for every question assessed.

**Table 2:** Topic distributions at different timepoints during the COVID-19 pandemic.

Question	T	Topic	Dec 2020	June 2021	Jan 2022	*χ*^2^
Why do you think more restrictions are needed?	T1	More needs to be done to protect the NHS, frontline workers, and the most vulnerable	16.1 (*N* = 973)	17.2 (*N* = 293)	10.7 (*N* = 231)	473.4, *p* = 2.0857E-89
	T2	Infection, transmission, hospitalisation and death rates are rising	10.1 (*N* = 611)	11.4 (*N* = 194)	27.1 (*N* = 583)	
	T3	Rules need to be stricter, clearer and better enforced	18 (*N* = 1,086)	15 (*N* = 256)	12.5 (*N* = 269)	
	T4	Lockdown should have been more complete and prompter	14.1 (*N* = 853)	19.5 (*N* = 333)	14.2 (*N* = 307)	
	T5	The government’s response to COVID-19 should be more proactive and stricter	17.6 (*N* = 1,064)	13.6 (*N* = 232)	12 (*N* = 259)	
	T6	Too many people do not follow COVID-19 rules and guidelines	13.8 (*N* = 834)	10.4 (*N* = 178)	16.2 (*N* = 350)	
	T7	Stronger, clearer restrictions are required to prevent the spread of the virus	10.2 (*N* = 619)	12.9 (*N* = 221)	7.2 (*N* = 156)	
Why do you think restrictions are unjustified?	T1	The physical, mental and economical toll due to COVID-19 policies are not justified by their impact on the pandemic	19.2 (*N* = 380)	26.7 (*N* = 414)	10.7 (*N* = 231)	137.3, *p* = 4.502E-24
	T2	The government is inconsistent and indecisive in coming up with and applying COVID-19 rules	13.9 (*N* = 274)	18.8 (*N* = 291)	27.1 (*N* = 583)	
	T3	Lockdowns and other measures are ineffective, either because more should have been done sooner, or because they basically do not work	14.6 (N = 289)	9.8 (N = 152)	12.5 (N = 269)	
	T4	COVID-19 policies are often illogical, contradictory and unfair across different contexts (e.g., pubs, schools, supermarkets, small businesses	18.5 (*N* = 365)	17.8 (*N* = 277)	14.2 (*N* = 307)	
	T5	The toll on young people’s health, wealth and opportunity from lockdown is not justified by its effect on disease burden	18.9 (*N* = 373)	10.8 (*N* = 167)	12 (*N* = 259)	
	T6	Restrictions should be focused on the vulnerable, allowing the majority to get on with life for their and the economy’s benefit	15 (*N* = 297)	16.2 (*N* = 251)	16.2 (*N* = 350)	
Why do you think the government has ulterior motives?	T1	The government seems to prioritise COVID-19 policies that gain favourable opinion of businesses and the public resulting in slow and inconsistent decision-making	18.8 (*N* = 872)	25.7 (*N* = 729)	18.9 (*N* = 778)	116.7, *p* = 3.510E-20
	T2	The government has not been transparent/has been dishonest with the public, and has ignored the advice of experts	19.7 (*N* = 914)	19.8 (*N* = 561)	24.5 (*N* = 1,007)	
	T3	The economy has been prioritised over public health	22.5 (*N* = 1,044)	20.1 (*N* = 571)	19.8 (*N* = 815)	
	T4	The government is using COVID-19 as a shield to conceal its true plans or failures, especially with regards to Brexit	13.9 (*N* = 644)	11.2 (*N* = 317)	16.2 (*N* = 666)	
	T5	Government ministers have used the pandemic for personal gain and to unfairly reward friends and supporters, especially through award of contracts	25.3 (*N* = 1,174)	23.2 (*N* = 659)	20.7 (*N* = 851)	
Why do you think the media is hiding things?	T1	The media tends to share partial and decontextualised information based on ambivalent reasons	10.3 (*N* = 352)	9.4 (*N* = 208)	9.8 (*N* = 255)	29.4, *p* = 0.002
	T2	The media seems to focus on the negative, sensational storeys, often with profit as their primary interest	15.6 (*N* = 533)	15.2 (*N* = 336)	16.1 (*N* = 419)	
	T3	The media does not encourage critical discourse backed by scientific evidence about the pandemic and the government’s handling of it	10.1 (*N* = 344)	12.8 (*N* = 282)	14.6 (*N* = 381)	
	T4	The media does not present impartial news because it is influenced by the politics, the global economy and their investors	20.5 (*N* = 702)	20.6 (*N* = 455)	19.9 (*N* = 517)	
	T5	The media is unreliable for various reasons	14.4 (*N* = 493)	13.8 (*N* = 305)	11.4 (*N* = 297)	
	T6	The media often has their own biases and agendas, and do not present enough global or alternative views	15.4 (*N* = 526)	14.8 (*N* = 327)	15 (*N* = 390)	
	T7	COVID-19-related statistics are perceived as misleading or lacking	13.7 (*N* = 470)	13.4 (*N* = 297)	13.1 (*N* = 342)	
Why would you refuse the COVID-19 vaccination?	T1	The testing and rollout of the vaccine was too rushed for some people, and others are medically exempt	21.6 (*N* = 283)	15.9 (*N* = 33)	11.2 (*N* = 30)	54, *p* = 4.308E-10
	T2	People are unsure about taking either vaccines in general or the COVID-19 vaccine, and would prefer not to	19 (*N* = 249)	18.8 (*N* = 39)	25.7 (*N* = 69)	
	T3	The benefits of the vaccine are not worth its perceived risk or efficacy	22.6 (*N* = 296)	19.8 (*N* = 41)	9.7 (*N* = 26)	
	T4	Vaccine testing was too quick, and could not evaluate long-term side effects	36.7 (*N* = 481)	45.4 (*N* = 94)	53.5 (*N* = 144)	
Why would you refuse the COVID-19 vaccination?	T1	Human interference with animal biosphere has encouraged zoonosis	16.3 (*N* = 648)	14.7 (*N* = 510)	14.2 (*N* = 537)	430, *p* = 9.223E-87
	T2	The rapidity and severity of the pandemic coupled with opaque investigations, makes people unsure of the virus’s origin	11.5 (*N* = 457)	10.4 (*N* = 362)	9.5 (*N* = 357)	
	T3	COVID-19 is a result of humans mistreating, eating and interfering with animals in their habitats	18.5 (*N* = 735)	31.9 (*N* = 1,107)	35.5 (*N* = 1,341)	
	T4	A lack of hygiene regulations, most importantly in food markets, has created conditions for Zoonosis	14.3 (*N* = 973)	10.7 (*N* = 370)	10.6 (*N* = 402)	
	T5	The virus was man-made in China, possibly as a biological weapon, and it was either accidentally or deliberately leaked	20.7 (*N* = 821)	21.3 (*N* = 737)	21.6 (*N* = 816)	
	T6	COVID-19 was created by humans, most likely in a lab, and was either deliberately or accidentally released	18.6 (*N* = 739)	11 (*N* = 381)	8.6 (*N* = 323)	

Distributions of opinions of participants surveyed at the different timepoints are presented alongside chi-squared statistics (χ^2^); T, Topic.

Respondents who indicated more or less restrictions were needed were triaged into two separate LDAs. Free text responses from those who endorsed more restrictions were best explained by an LDA model with 7 latent topics. On thematic analysis of top 20 defining opinions for each topic in December 2020, these were labelled, in the order of their prevalence at that time, as follows: “Rules need to be stricter, clearer and better enforced” (18%), “The government’s response to COVID-19 should be more proactive and stricter” (17.6%), “More needs to be done to protect the NHS, frontline workers, and the most vulnerable” (16.1%), ‘Lockdown should have been more complete and prompter’ (14.1%), “Too many people do not follow COVID-19 rules and guidelines” (13.8%), “Stronger, clearer restrictions are required to prevent the spread of the virus” (10.2%) and “Infection, transmission, hospitalisation and death rates are rising” (10.1%). The prevalence of these topics significantly changed over time (*χ*^2^ = 473.4, *p* < 0.001). The highest percentage change between December 2020 and January 2022 was for the topic related to infections, transmission, hospitalisation and death rates, rising concerns which increased substantially from 10.1% in December 2020 to 27.1% in January 2022.

Conversely, free text responses for those who indicated that restrictions were unjustified were best explained by an LDA model with 6 topics, which were labelled, in the order of their prevalence in December 2020 as follows: “The physical, mental and economical toll due to COVID-19 policies are not justified by their impact on the pandemic”(19.2%), ‘The toll on young people’s health, wealth and opportunity from lockdown is not justified by its effect on disease burden’(18.9%), “COVID-19 policies are often illogical, contradictory and unfair across different contexts (e.g., pubs, schools, supermarkets, small businesses)” (18.5) “Restrictions should be focused on the vulnerable, allowing the majority to get on with life for their and the economy’s benefit”(15%), “Lockdowns and other measures are ineffective, either because more should have been done sooner, or because they basically do not work” (14.6%) and (%), “The government is inconsistent and indecisive in coming up with and applying COVID-19 rules” (13.9%). The change in the prevalence of topics across time was significant (*χ*^2^ = 137.3, *p* < 0.001), with the greatest increase being for government inconsistencies about the restrictions, which rose from 13.9% in December 2020 to 27.1% in January 2022.

An LDA with 5 topics best explained the reasoning underlying people’s assumption of ulterior motives behind governmental action. Based on December 2020 data, in the order of their prevalence, these topics were labelled as follows: “Government ministers have used the pandemic for personal gain and to unfairly reward friends and supporters, especially through award of contracts.” (25.3%), “The economy has been prioritised over public health” (22.5%), “The government has not been transparent/has been dishonest with the public, and has ignored the advice of experts” (19.7%), “The government seems to prioritise COVID-19 policies that gain favourable opinion of businesses and the public resulting in slow and inconsistent decision-making” (18.8%), and “The government is using COVID-19 as a shield to conceal its true plans or failures, especially with regards to Brexit.” (13.9%). The change in the prevalence of topics across time was significant (*χ*^2^ = 116.7, *p* < 0.001). The most prevalent opinion in January 2022 was that the government has been dishonest with the public and ignored advice of experts (24.5%).

An LDA model with 7 topics best explained why people believed the media is hiding things. Based on December 2020 data, these topics were labelled, in the order of their prevalence, as follows: “The media does not present impartial news because it is influenced by the politics, the global economy and their investors” (20.5%), “The media seems to focus on the negative, sensational storeys, often with profit as their primary interest” (15.6%), “The media often has their own biases and agendas, and do not present enough global or alternative views” (15.4%), “The media is unreliable for various reasons” (14.4%), “COVID-19-related statistics are perceived as misleading or lacking” (13.7%), “The media tends to share partial and decontextualised information based on ambivalent reasons” (10.3%), and “The media does not encourage critical discourse backed by scientific evidence about the pandemic and the government’s handling of it” (10.1%). The change in prevalence of these topics across time was significant (*χ*^2^ = 29.4, *p* = 0.002). As time passed, the most substantial shift was in the proportion of respondents who considered the media did not encourage critical discourse: from 10.1% in December 2020 to 14.6% in January 2022.

Only a small proportion of people in our sample indicated that they would refuse the COVID-19 vaccination. In this case their reasoning was best explained by an LDA model with 4 topics which were, in order fo their prevalence, labelled as follows: “Vaccine testing was too quick, and could not evaluate long-term side effects” (36.7%), “The benefits of the vaccine are not worth its perceived risk or efficacy” (22.6%), “The testing and rollout of the vaccine was too rushed for some people, and others are medically exempt” (21.6%) and “People are unsure about taking either vaccines in general or the COVID-19 vaccine, and would prefer not to” (19%). The change in prevalence of these topics across time was significant (*χ*^2^ = 54, *p* < 0.001). The latter topic showed an increase from 36.7% in December 2020 to 53.5% in January 2022.

Lastly, people had a range of opinions concerning the origins of COVID-19. These were explained best by an LDA with 6 topics. These were labelled, in order of their prevalence, as follows: “The virus was man-made in China, possibly as a biological weapon, and it was either accidentally or deliberately leaked” (20.7%), “COVID-19 was created by humans, most likely in a lab, and was either deliberately or accidentally released” (18.6%), “COVID-19 is a result of humans mistreating, eating and interfering with animals in their habitats” (18.5%), “Human interference with animal biosphere has encouraged zoonosis” (16.3%), “A lack of hygiene regulations, most importantly in food markets, has created conditions for Zoonosis” (14.3%) and “The rapidity and severity of the pandemic coupled with opaque investigations, makes people unsure of the virus’s origin” (11.5%). The change in prevalence of these topics across time was significant (*χ*^2^ = 430, *p* < 0.001). The most prevalent topic in January 2022 was that the virus is the result of improper treatment of animals and interference with their habitats (35.5%), and the least prevalent, that it was created by humans and deliberately/accidentally released from a lab (8.6%).

#### 3.3.2. Topics covary substantially with population factors

**Figure 5:**
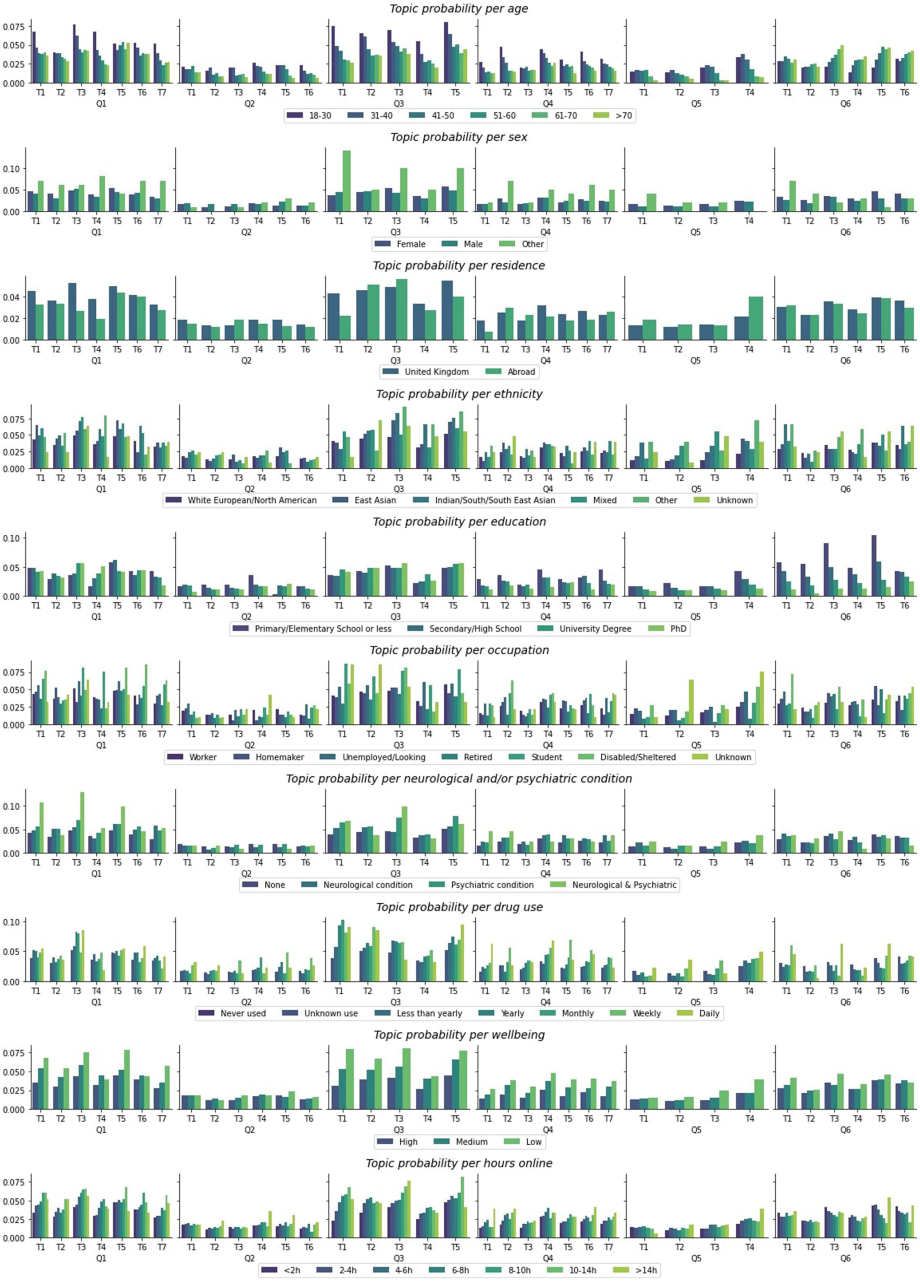
Topic probability distribution for participants of different sociodemographic characteristics. Probabilities are illustrated at population level, out of the total number of people surveyed at all timepoints; T, Topic.

Finally, we tested the hypothesis that the prevalence of scepticism topics would vary substantially across different segments of society. Specifically, we calculated probability distributions of best fitting topics within each factor level across all timepoints. Note – here probabilities are calculated for December 2020 (whereas changes over time are illustrated in Supplementary Figures S6, S7) for the whole population, not just those who endorsed statements that lead to free text explanations being requested in order to capture the overall probability that a member of a certain group subscribes to a certain topic. Chi-squared tests confirmed statistically significant variability for all topic prevalence differences across sociodemographics factors (Table 3 and Supplementary Table S5) (Figure 5).

**Table 3:** Topic probabilities co-vary with sociodemographic factors.

Factor	More restrictions needed		Restrictions are not justified		The government has ulterior motives		The media is hiding things		Refusing the vaccine		COVID-19 has unnatural origins	
	*χ*^2^	*p*-value	*χ*^2^	*p*-value	*χ*^2^	*p*-value	*χ*^2^	*p*-value	*χ*^2^	*p*-value	*χ*^2^	*p*-value
Age	145	***	61.6	***	56.5	***	104.4	***	41.3	***	141.6	***
Sex	90.3	***	103.1	***	49.7	***	64	***	13.6	*	90.1	***
Residence	15.6	*	5.4	0.369	9.5	*	6.5	0.367	7.5	0.058	14.1	*
Ethnicity	42.2	0.22	39.9	0.107	30.3	0.175	40.1	0.292	31.3	*	111.8	***
Education	120.3	***	33.6	**	45.3	***	59.8	***	8.1	0.525	198.6	***
Occupation	128.3	***	57.1	**	52.2	***	62.6	**	39	**	213.7	***
Neuropsychiatric status	12.4	0.823	24.4	0.059	23.7	*	23.9	0.158	4.9	0.841	57.9	***
Pre-pandemic drug use frequency	92.9	***	47.9	*	89.2	***	66.3	**	35.6	**	140	***
Mood	15.4	0.221	22.9	*	11.5	0.175	35.2	***	2.1	0.91	47.5	***
Time spent online	254.9	*	164.2	0.079	169	0.076	240.9	*	96.3	0.079	316.9	***

The highest probabilities for endorsing a certain topic are presented for different demographics below.

The highest probabilities we observe for certain sub-populations to subscribe to a specific topic are for those who identify as neither male or female to justify their scepticism about the government because “*The government seems to prioritise COVID-19 policies that gain favourable opinion of businesses and the public resulting in slow and inconsistent decision-making*,” for those not educated past primary school level to consider the Sars-Cov-2 virus to be the result of “*COVID-19 is a result of humans mistreating, eating and interfering with animals in their habitats*” or *“The virus was man-made in China, possibly as a biological weapon, and it was either accidentally or deliberately leaked,”* for those suffering with both a neurological and a psychiatric condition to consider the level of restrictions at that time unjustified because of “*More needs to be done to protect the NHS, frontline workers, and the most vulnerable*,” “*Rules need to be stricter, clearer and better enforced*,” “*The government’s response to COVID-19 should be more proactive and stricter”* and to be sceptical about the government mostly because “*The economy has been prioritised over public health*.” Finally, some of the highest probabilities were also observed for illicit drug users to distrust the government specifically because “*The government seems to prioritise COVID-19 policies that gain favourable opinion of businesses and the public resulting in slow and inconsistent decision-making*,” “*The government has not been transparent/has been dishonest with the public, and has ignored the advice of experts*,” “*Government ministers have used the pandemic for personal gain and to unfairly reward friends and supporters, especially through award of contracts.”*

Of notable mentions, other demographic sub-categories also exhibit notable variability in using certain justifications for their scepticism, which are important to consider given the global context. For example, we observe differences in how different ethnic groups justify a variety of views. Most strikingly, all ethnic minorities had higher probabilities than white people to refuse the COVID-19 vaccination because of either topics – “*The testing and rollout of the vaccine was too rushed for some people, and others are medically exempt*,” “*People are unsure about taking either vaccines in general or the COVID-19 vaccine, and would prefer not to*,” “*The benefits of the vaccine are not worth its perceived risk or efficacy*,” “*Vaccine testing was too quick, and could not evaluate long-term side effects,*” with the latter having the highest probability. Certain ethnic minorities such as East Asians, Indian/South East Asians and Others also had the highest probability out of all ethnic groups to be sceptical about the government because of *“The economy has been prioritised over public health”* and *“Government ministers have used the pandemic for personal gain and to unfairly reward friends and supporters, especially through award of contracts.”* Different occupations also meant higher probabilities for certain topics. For example, disabled individuals had higher probabilities to consider the level of restrictions unjustified because of “*More needs to be done to protect the NHS, frontline workers, and the most vulnerable*,” “*The government’s response to COVID-19 should be more proactive and stricter*,” “*Too many people do not follow COVID-19 rules and guidelines*,” distrust the government because of “*The economy has been prioritised over public health*” and refuse the vaccine because “*People are unsure about taking either vaccines in general or the COVID-19 vaccine, and would prefer not to*,” and believe the origins of the virus are “*Human interference with animal biosphere has encouraged zoonosis*.” Students on the other hand had high probabilities to justify considering the restrictions inadequate because of “*Lockdown should have been more complete and prompter*” and be sceptical about the government because of “*The government seems to prioritise COVID-19 policies that gain favourable opinion of businesses and the public resulting in slow and inconsistent decision-making*,” “*The economy has been prioritised over public health*,” “*Government ministers have used the pandemic for personal gain and to unfairly reward friends and supporters, especially through award of contracts.*”

In certain instances notable monotonic trends were also observed in our data. For example, in the case of age, younger individuals (18–30 years old) had higher probability for all topics related to the level of restrictions, distrusting the government, media and vaccines, and these probabilities decreased monotonically for older ages. An inverse trend was observed for the justifications pertaining to the origins of the virus, specifically related to topics “*COVID-19 is a result of humans mistreating, eating and interfering with animals in their habitats,”* “*A lack of hygiene regulations, most importantly in food markets, has created conditions for Zoonosis*,” “*The virus was man-made in China, possibly as a biological weapon, and it was either accidentally or deliberately leaked*,” “*COVID-19 was created by humans, most likely in a lab, and was either deliberately or accidentally released*” where the older an individual, the higher probability they had to justify the origins of Sars-Cov-2 via unnatural means. In regards with education, a monotonic relationship was also observed for a number of topics. For example, the lower the education levels of an individual, the higher the probability to think “*Lockdown should have been more complete and prompter.*” Conversely, the lower the education levels, the less probable individuals were to consider “*Stronger, clearer restrictions are required to prevent the spread of the virus*,” to give any of the justifications pertaining to distrusting the media, refusing the vaccine or believing Sars-Cov-2 has unnatural origins. In regards with mood, we also observe that the more negative mood someone has, the higher the probability of them to subscribe to any of the topics pertaining to the level of restrictions being unjustified and being sceptical about the government and media. Finally, time online was also part of identified monotonic trends. In relation to the level of restrictions being inappropriately low, the more time someone spends online the higher the probability to “*More needs to be done to protect the NHS, frontline workers, and the most vulnerable*,” “*Infection, transmission, hospitalisation and death rates are rising*,” “*Rules need to be stricter, clearer and better enforced*,” “*Lockdown should have been more complete and prompter*,” but also to be sceptical about the government and justify it based on “*The government seems to prioritise COVID-19 policies that gain favourable opinion of businesses and the public resulting in slow and inconsistent decision-making*,” “*The economy has been prioritised over public health*.”

## 4. Discussion

As expected, our results highlighted a significant relationship whereby the substantial minority of people who were sceptical of the mainstream government and media narrative about COVID-19 themes were also less compliant with key aspects of rules and advice during the pandemic. Perhaps unsurprisingly, this relationship was particularly pronounced amongst people who considered the restrictions unjustified. Notably, in some key respects the proportion of sceptical opinions appeared to have increased as the pandemic progressed, e.g., in relation to governmental ulterior motives and the origins of the virus, which were matched with a corresponding change in compliance. Concomitantly, social distancing significantly decreased and more people only wore masks because they had to. Some components in these changes in compliance, such as the dramatic reduction in respondents no longer avoiding leaving the house by January 2022, will have reflected changes in context and government advice. However, a causal relationship between opinion and behaviour has been conceptualised in previous studies (Braun and Clarke, 2006; Bargain and Aminjonov, 2020; Freeman et al., 2022; Wright et al., 2022b) and is likely also to be a contributing factor. Moreover, the results accord closely with the view that the general public had an increasingly negative view of government action and advice as the pandemic progressed.

By using an approach whereby individuals could first indicate whether they endorsed sceptical comments about the government and media narrative on broad pandemic themes and then asking them to justify that scepticism, we were able to identify the views that people held in a manner that gave them scope to respond in an unconstrained manner within targeted themes. Our results demonstrate that attributing non-compliance to a generic level of scepticism is an oversimplification. This is because the sceptical opinions that people hold within any given pandemic theme, e.g., handling of the pandemic by bodies of authority, vaccines and the origins of the virus, are diverse. These opinions can be modelled as finer grained topics and can be described as spanning from perceptions of incompetence and inappropriate prioritisation on the part of government to beliefs in nefarious intentions and full blown conspiracy theories. This diversity of sceptical views presents a non-trivial challenge for any attempt at improving compliance through rational argument.

Offering a possible handle on this diversity, the specific reasons underlying sceptical opinion topics all covaried significantly with population demographic variables. For example, consistent with previous studies (Duplaga, 2020; Georgiou et al., 2020; Parsons and Wiggins, 2020; Allington et al., 2021; Enria et al., 2021; Freeman et al., 2022), ethnic minorities did have higher probabilities across the board to subscribe to topics pertaining to the refusal of the vaccine compared to those who were White Europeans/North Americans. However, by looking at the specific reasoning provided by these minorities to refuse the vaccine, rather than simply their refusal, we have a chance to understand where the scepticism is coming from and address that in practise with targeted and nuanced conversation. Examining the topics, we learn that the reason characterised by the highest probability for the ethnic minorities for being sceptical pertains to a lack of evaluation of long-term side effects. Higher level of scepticism were also observed amongst those who did not complete education past primary school level,most strikingly in relation to holding alternative views (such as the potential of the virus being a bioweapon, or a product of humans mistreating animals and destroying their habitat) about the origins of the Sars-Cov-2 virus, but not in other instances. Conversely, people of higher education were more likely to subscribe to topics pertaining to the government not doing enough and having questionable motivations, with those being educated at PhD level being most likely to endorse the need for stricter restrictions and justify their scepticism against the government based on perceptions of officials having used the pandemic for personal gain through the award of lucrative contracts. These observations highlight once again that it is overly simplistic to simply ascribe general levels of scepticism to specific subsets of the population, but what is necessary in the global discourse is to consider how the background of an individual shapes the very specific aspects of current policy or discourse that they would be most sceptical about. This considered, one might argue that there is benefit to characterising the most prevalent reasons for people being sceptical and targeting counterarguments based on demographic, or other population factors not limited to those captured in the present analysis. However, given that the topics appear to vary substantially but not in an absolute manner, it might be more fruitful where possible, to characterise each individual’s views based on free text against models of the most prevalent views and to target counterarguments accordingly.

Adding to the complexity, we observed that sceptical opinion topics also significantly evolved over time. Despite overall statistical significance pertaining to changes over time, which is expected at the scale of the data analysed, some changes were subtle, e.g., the proportion of people who stated the government ignored expert advice rose from ~21% to ~25% between December 2020 to ~30% in January 2022. Other changes were more substantial, e.g., the proportion of people stating that COVID-19 was caused by humans mistreating animals/nature rose from ~18% to ~35% in the same timeframe. Taken together, the fact that the sceptical views people hold vary with sociodemographic factors and are dynamic across time presents a major challenge for any attempts to improve compliance through rational argument directed to the whole population at once, in the same style. Beyond sociodemographics, it is likely that the change in topic prevalence that we observe is also rooted, to a certain degree, in the swift changes in policy during the first few years of the COVID-19 pandemic. As such, views about the adequate levels of restrictions, for example, could have fluctuated in response to guidelines present at the time of assessment. This hypothesis seems likely. Drawing a parallel to a study published during 2021 tracking COVID-19 policies in 180 countries including the United Kingdom reveals that by the end of 2020, around Christmas time, policies became stricter world-wide (Hale et al., 2021). This is potentially reflecting why a large proportion of our respondents justified their scepticism about restrictions at that time by calling out lockdowns as ineffective and condemning the negative effect of strict policies on young people’s health, and why these justifications were more prevalent then than during any of the subsequent timepoints of assessment.

There are also obvious technical challenges to the approach we propose due to the requirement for ongoing data collection in order to keep models up to date. In this respect, it is notable that a number of previous studies have used free text analysis to investigate public opinion in the context of the COVID-19 pandemic, primarily with social media data. However, these studies tend to report topics that have less granularity than those presented here. For example, one study using LDA to model Twitter responses focused on understanding the common themes underlying anxiety in the general population, compared this across nations and identified political polarisation and governmental incompetence as common relevant topics (Kwon and Park, 2022). Another study that used LDA to model Twitter data in the United Kingdom to examine discussions and concerns of the population during the pandemic highlighted lockdown and government as prominent topics (Cheng et al., 2021). Though social media studies pose advantages – such as enabling the passive collection and modelling of data in a near continuous fashion – the lesser granularity presents a major methodological limitation. Here, by focusing the free text response on specific themes with periodic citizen science sampling, we provide a greater level of granularity that captures the different prevalent opinions that people hold – what for most previous studies is a topic, in our study it is rendered as a theme having finer grained topics. This in combination with a higher level of certainty and detail regarding demographics and lack of bots (each person had to complete tests and a questionnaire in this study) mean we have higher confidence in the modelled topics and their relationships to demographic factors being reasonably representative of the general public than what we could infer from social media posts (Enria et al., 2021; Tsao et al., 2021; Gollwitzer et al., 2022; Wright et al., 2022b).

Some limitations should be considered. Though large, our sample is not proportionally representative of the United Kingdom population; we highlight a bias toward older adults and those holding a university degree, which was amplified over time. However, the same method that we have used here could readily be applied with even more rigour in the context of random or representative sampling epidemiological surveys, where there is yet greater certainty regarding peoples’ identities. Nonetheless, we note that the population we surveyed is inclusive of a diversity of people such that associations of distrust and beliefs can be modelled across a comprehensive set of population variables with considerable sensitivity even to small associations. Moreover, a considerable number of individuals from different minority groups who are typically less likely to volunteer to take part in government and mainstream surveys – such as illicit substance users and persons suffering with neurological or psychiatric conditions – are represented within our study. We posit that this inclusivity reflects that our cohort was recruited as a citizen science project primarily through the BBC, that is, as opposed to governmental communication through official survey platforms that some subsets of the population might be less inclined to respond to. In keeping with all sampling methods, it is the case that we likely underestimate levels of distrust and non-compliance in the general population, as those who are distrustful are likely to be more sceptical of taking part in survey-based studies (Wright et al., 2022a,b). However, we are able to model prevalence across many relevant demographic factors and the use of citizen science provides a happy compromise, whereby public opinion is efficiently surveyed from the general population repeatedly at large scale with relatively little cost. Relatedly, people who do engage with surveys, studies looking at the difference between self-reported and actual compliance (although, United States-based) have found self-report to be a reliable measure for studying compliance (Gollwitzer et al., 2022).

We also included responses from people who live abroad and may not feel the direct effects of the local policies, and hence have somewhat skewed opinions. However, given the low proportions (at most 6.5% of the total sample at each timepoint) this is unlikely to have driven our results at large. It also is important to highlight that only a minority of individuals completed all of the analysed timepoints. Notably though, a broad range of demographic profiles were represented across these timepoints, and our results were consistent with past findings from studies employing representative samples. Since the representation of different population characteristics was vast in our sample, it is also possible that this reflected itself more profoundly in the way that respondents interpreted the prompt (free text questions) to begin with. However, we did not curate data in order to reflect our (researchers) interpretation of the questions as absolute, thereby allowing the themes to emerge from a diverse set of perspectives pertaining to key pandemic related themes.

Importantly, we note that both scepticism and compliance are likely to be explainable through associations with variables not limited to those we surveyed. For example, at the time when the present data were collected, political affiliation was not part of the survey, and consequently we do not have a handle of this variable to account for. It is not unlikely that if a skewed representation of political affiliations is part of the sample the natural language models are trained on, this could influence the resulting themes to be more representative of that orientation. Additionally, other factors not captured within the present assessment might also be relevant to understanding how scepticism varies across the general population such as income, strength of personal relationships, conflict at home and personality structure.

On a methodological level, there are two mentions to be made about both the survey and the natural language processing algorithm. First, we did not use established scales to measure either scepticism and non-compliance, and therefore cannot be certain to what degree we overlap with established constructs in the field. The questionnaires we employed were bespoke for the present study. However, insofar as this approach is useful in allowing us to model responses pertaining to very specific questions of interest related to the pandemic, it cannot be used to infer general levels of scepticism present in the sample beyond what was surveyed, or consider that distrusting the mainstream COVID-19 narrative is indicative of conspiratorial thinking more broadly. Scepticism and conspiracy are themselves established nuanced constructs and form the basis of specific fields of research. Future studies might benefit from including scales of population distrust in the beliefs, intentions, and behaviour of bodies of authority with tailored questions while at the same time assessing the degree of conspiratorial thinking of individuals with validated scales such as the Generic Conspiracist Beliefs Scale (Brotherton et al., 2013), and analyse them in conjunction with free text data. This can determine whether those who nurture conspiracy theories more generally or rather particular aspects of distrust (distrusting intentions, distrusting behaviours, distrusting beliefs) overlap, whether they are more likely to be non-compliant, and importantly, why. Ultimately though, the problem of discerning the exact causal links from associations between opinions and behaviours needs to be considered in the light of endogeneity characteristic to these variables not only in the present study but also beyond, regardless of the level of granularity employed (Podsakoff et al., 2012).

Secondly, we used LDA, one of the oldest and most established, and proven to work well topic modelling techniques (Blei et al., 2003). Our thematic analysis of LDA topics highlight that many people are distrustful of the government and media and that a smaller minority subscribes to views that may be considered conspiratorial, for example, considering the SARS-CoV-2 virus to be an intentionally-released bioweapon. A key strength of the current study is that these topics were identified in a data-driven manner, that is, as opposed to being selected from researcher-defined options. This means there is less bias in our estimates of their relevance and prevalence – it has previously been reported that fixed answer surveys can act as self-fulfilling prophecies (Butter and Knight, 2015) where respondents echo the researchers’ views. However, a diversity of variants on topic modelling methodology are available. Future work should explore application of alternative topic modelling methods and coherence algorithms. Nonetheless, the topics derived using the approach applied here have clear validity, being interpretable and correlating robustly with population variables in a manner that makes intuitive sense, whilst both replicating and extending findings from past studies. When analysed alongside sociodemographic and lifestyle factors they provide key insights for shaping targeted messaging. These outputs have potential applications in crafting and shaping public policies, interventions and campaigns, and their usefulness should be investigated in upcoming studies. For example, these methods could be used not only mapping the major themes of distrust or scepticism circulating in the general population as we did here, but specifically using sociodemographic or individual-based approaches informed by these data-driven themes to communicate with members of the general population in order to solve disputes, misunderstandings or divergence of opinions. The efficacy of these approaches should also be investigated in future studies.

In summary, the diverse and shifting nature of sceptical opinions that associate with non-compliance presents a major challenge to any efforts to improve compliance through rational argument. The COVID-19 pandemic has presented a unique opportunity to examine the relationships between the public and the bodies of authority at a finer grain than has been previously possible. However, the general principles that have been observed have relevance to other future pandemics, crisis situations, and more generally to the interplay between opinions about the government and media, and population behaviour. It seems reasonable to suggest that aspects of the methodology implemented here could be applied for this type of purpose, since it is reproducible and applicable to any free text data in response to targeted questions (and not only). Specifically, the combination of large-scale citizen science survey data and natural language based machine learning can be used to rapidly identify the currently most prevalent opinions that underlie disagreements, distrust, conspiracy theories and ultimately non-compliance. These may then be addressed in a manner that is tailored either to the individual’s opinions, as referenced against the broader corpus of free text topics, or at a coarser grain by mapping those topics across sociodemographic variables. Ultimately, understanding the underlying rationale behind the behaviours exhibited by diverse individuals or groups may hold significant potential in effectively addressing the communication disparities that exist between them.

## Data availability statement

Data presented are part of a longitudinal study that is still ongoing, and can only be shared via institutional agreements in compliance with local GDPR guidelines. Data sharing inquiries should be directed to Prof. Adam Hampshire: a.hampshire@imperial.ac.uk.

## Ethics statement

This study was run in accordance with the Helsinki Declaration of 1975, as revised in 2008. All procedures were approved by the Imperial College Research Ethics Committee (17IC4009). The patients/participants provided their electronic informed consent to participate in this study.

## Author contributions

MB, AJ, and AH: conceptualization. MB, AH, and DK: methodology. MB: investigation. MB, AL, and DK: visualisation. AH: supervision. WT, PH, and AH: software. MB, DK, DG, DC, and AL: writing—original draft. MB, DK, DG, AL, WT, AJ, DC, PH, and AH: writing—review & editing. All authors contributed to the article and approved the submitted version.

## Funding

MB is supported by the Medical Research Council Doctoral Training Programme at Imperial College London. DK is supported by the Vice Chancellor Studentship at the University of Surrey. DG is supported by the Department of Brain Sciences, Imperial College London. AL is supported by the UK Research and Innovation Centre for Doctoral Training in AI for Healthcare http://ai4health.io (grant number EP/S023283/1) and the UK MS Register. DC is supported by the UK Dementia Research Institute: Care Research & Technology Centre. WT is supported by the EPSRC Centre for Doctoral Training in Neurotechnology. PH is, in part, supported by the National Institute for Health Research (NIHR) Biomedical Research Centre at South London and Maudsley NHS Foundation Trust and King’s College London. AJ is supported by the Queen Square Multiple Sclerosis Centre at University College London. AH is supported by the Biomedical Research Centre at Imperial College London.

## Conflict of interest

AH is owner and director of Future Cognition LTD and H2 Cognitive Designs LTD, which support online studies and develop custom cognitive assessment software, respectively. PH is owner and director of H2 Cognitive Designs LTD and reports personal fees from H2 Cognitive Designs LTD outside the submitted work.

The remaining authors declare that the research was conducted in the absence of any commercial or financial relationships that could be construed as a potential conflict of interest.

## Publisher’s note

All claims expressed in this article are solely those of the authors and do not necessarily represent those of their affiliated organizations, or those of the publisher, the editors and the reviewers. Any product that may be evaluated in this article, or claim that may be made by its manufacturer, is not guaranteed or endorsed by the publisher.
