# Supplementary material for: Mapping the sociodemographic distribution and self-reported justifications for non-compliance with COVID-19 guidelines in the United Kingdom
Source: Front Psychol. 2023 Jul 19;14:1183789. doi: 10.3389/fpsyg.2023.1183789 (PMC10395087; doi:10.3389/fpsyg.2023.1183789)
Supplement: Supplementary file 1 [file Table_1.pdf]

Supplementary Material

Mapping the sociodemographic distribution and self-reported justifications for non-compliance with COVID-19 guidelines in the UK

Maria Bălăeț*, Danielle L. Kurtin, Dragos C. Gruia, Annalaura Lerede, Darije Custovic, William Trender, Amy E. Jolly, Peter J. Hellyer, Adam Hampshire

*Corresponding author. Email: m.balaet17@imperial.ac.uk

This file includes:

Supplementary Text – Full questionnaire items, Further topic modelling explanations

Figs. S1 to S9

Table S1 , S6, S7

Other Supplementary Materials for this manuscript include the following separate files:

Table S5

Supplementary Text

<u>Full questionnaire items</u>

Part 1 – Demographics

1. How old are you?

From 0 to 100

2. What sex have you been assigned?

a. Male

b. Female
c. Other

3. To ensure we have a representative sample of the population, please indicate your ethnicity:

a. White or North American
b. East Asian
c. Indian/S/SE Asian
d. Mixed ethnicity
e. Other
f. Unknown

4. What is your residence?

a. United Kingdom
b. Abroad

5. What is your level of education?

a. Primary/elementary school or less
b. Secondary school/High school diploma
c. University Degree
d. PhD

6. What is your occupational status?

a. Worker
b. Student
c. Homemaker
d. Retired
e. Unemployed/looking for work
f. Disabled/unapplicable/sheltered employment
g. Unknown

Part 2 – Mental health and wellbeing

These items were scored on a continuous scale from 0 to 6 and 0 to 7 respectively. The scores were grouped in three categories for analysis purposes: high wellbeing (below 20), medium wellbeing (between 20 and 40) and low wellbeing (above 40).

The GAD-7 Questionnaire items of choice

1. Are you feeling nervous, anxious or on edge?

a. Never,
b. Almost never
c. Once or twice a week
d. Several times a week
e. Daily
f. Hourly
g. More Often

2. Are you not being able to stop or control worrying?

a. Never

b. Almost never
c. Once or twice a week
d. Several times a week
e. Daily
f. Hourly
g. More Often

3. Are you worrying too much about different things?

a. Never
b. Almost never
c. Once or twice a week
d. Several times a week
e. Daily
f. Hourly
g. More Often

4. Are you having trouble relaxing?

a. Never
b. Almost never
c. Once or twice a week
d. Several times a week
e. Daily
f. Hourly
g. More Often

5. Are you being so restless that it is hard to sit still?

a. Never
b. Almost never
c. Once or twice a week
d. Several times a week
e. Daily
f. Hourly
g. More Often

6. Are you becoming easily annoyed or irritable?

a. Never
b. Almost never
c. Once or twice a week
d. Several times a week
e. Daily
f. Hourly
g. More Often

7. Are you feeling afraid as if something awful might happen?

a. Never
b. Almost never
c. Once or twice a week
d. Several times a week
e. Daily
f. Hourly

g. More Often

The PHQ Questionnaire items of choice

1. How often do you have little interest or pleasure in doing things?

a. Never
b. Almost never
c. Once or twice a week
d. Several times a week
e. Daily
f. Hourly
g. More Often

2. How often are you feeling down or depressed?

a. Never
b. Almost never
c. Once or twice a week
d. Several times a week
e. Daily
f. Hourly
g. More Often

3. How often are you feeling tired or having little energy?

a. Never
b. Almost never
c. Once or twice a week
d. Several times a week
e. Daily
f. Hourly
g. More Often

4. How often do you have trouble concentrating on things, such as reading the newspaper or watching television?

a. Never
b. Almost never
c. Once or twice a week
d. Several times a week
e. Daily
f. Hourly
g. More Often

5. How often are you not being able to get to sleep or stay asleep?

a. Never
b. Almost never
c. Once or twice a week
d. Several times a week
e. Daily
f. Hourly
g. More Often

Part 4 – Additional Questions

1. On average, how many hours a day do you spend online?

Choice from 0 to 24

2. Have you been diagnosed with a neurological or psychiatric condition?

a. No
b. I have a neurological condition
c. I have a psychiatric condition
d. I have both a neurological and a psychiatric condition

3. How often did you use to take recreational drugs prior to the pandemic?

a. Never
b. Less than yearly
c. Yearly
d. Monthly
e. Weekly
f. Daily
g. Skip this question (unknown use)

Part 5 – Questions about opinion and compliance during the COVID-19 pandemic

Compliance questions

Compliance questions were scored on a continuous scale from 0 to 2. The total scores represented the compliance score. The vaccine questions was initially asked when vaccines were just starting to be rolled out.

1. Do you wear a mask in enclosed public spaces?

a. Yes = 0
b. Yes, but only because I have to = 0
c. No, I am exempt = 0 (because this is still complying)
d. No, I don’t think it makes a difference = 2

2. Do you avoid leaving the house due to Covid?

a. Yes, I am scared for my health = 0
b. Yes, I am shielding = 0
c. Yes, I don’t trust others to be sensible = 0
d. Yes, for other reasons = 0
e. No, not anymore = 1
f. No = 2

3. Have you followed the government guidelines about social distancing?

a. Yes = 0
b. Yes, most of the time = 1
c. I used to but I have given up = 1
d. No = 2

4. Do you use the NHS Covid-19 app?

a. Yes = 0
b. No, it doesn’t work on my phone = 0
c. No, I don’t want to be tracked = 1
d. No, I don’t trust what the government will do with that data = 1

5. When a vaccine is developed, would you get it?

a. Yes = 0
b. No = 2

Questions about counterfactual compliance

1. When a vaccine is developed, would you get it?

a. Yes = 0
b. No - Free text = 1

Questions about opinions

For some of the answers participants were also asked to provide their reasoning in free text form.

2. Do you feel that there are ulterior motives behind the government’s response to Covid?

a. Yes – Free text = 1
b. No = 0

3. Do you think that the media is hiding things from the public?

a. Yes– Free text= 1
b. No= 0

4. Do you think that Covid is a naturally occurring phenomenon?

a. Yes= 0
b. No – Free text= 1

5. Do you feel that current lockdown restrictions are justified?

a. Yes = 0
b. Yes, but more needs to be done – Free text= 1
c. No – Free text= 1

Question about information sources

1. What are your primary sources of information about the pandemic?

a. Word of mouth
b. TV News
c. Radio
d. Newspapers
e. Social media
f. Podcasts
g. Internet news sites
h. Governmental communications

Further topic modelling explanations - Coherence

We estimated the average coherence per number of topics, ranging from 1-30 for each question (Figure S3). Coherence measures assess whether topics are more or less internally consistent; therefore, the number of topics with the highest coherence (range from 4-7) was chosen for each question as the optimal number of topics.

Then, LDA was applied using the optimal number of topics illustrated as the peaks in Figure S3 for each question.
The probabilistic graphical model of LDA is represented in Figure S4 (adopted from Syed and Spruit 2017). The topic mixtures per opinion were obtained and the top 20 opinions and words per topic were ranked based on this. Finally, the top 20 opinions associated with each topic were used to interpret and label each topic.

Predicting individual answers to opinion and compliance questions from sociodemographic and lifestyle factors.

We performed binomial and multinomial logistic regression on the data using Python statmodels library (*Seabold et al., 2010*) in order to identify questionnaire variables predictive of people more likely to answer a question in a distrustful/noncompliant way. The questionnaire variables investigated include sociodemographic features (age, gender, occupation, education, ethnicity and country of residence), levels of substance use, neuropsychiatric status, wellbeing and number of hours spend online per day and primary source of news about the pandemic. All empty values were dropped, and dummy variables were computed for all categorical data including the predictors and the dependent variables. The predictor categories with the highest number of participants acted as reference categories and were removed from the models. We also checked for highly correlated variables and found that questions such as those asking about mask wearing and about socially distancing had very low numbers of responses in certain categories, so those were collapsed together and the questions entered binomial rather than multinomial regression models. For each model odds ratios were calculated to reflect odds compared to the removed highest scoring dummy. Effect size (Cohen's d) was calculated by dividing the beta coefficients calculated in the linear regression by the square root of the N times the standard errors. Figure S2 illustrates the results.

Fig. S1.

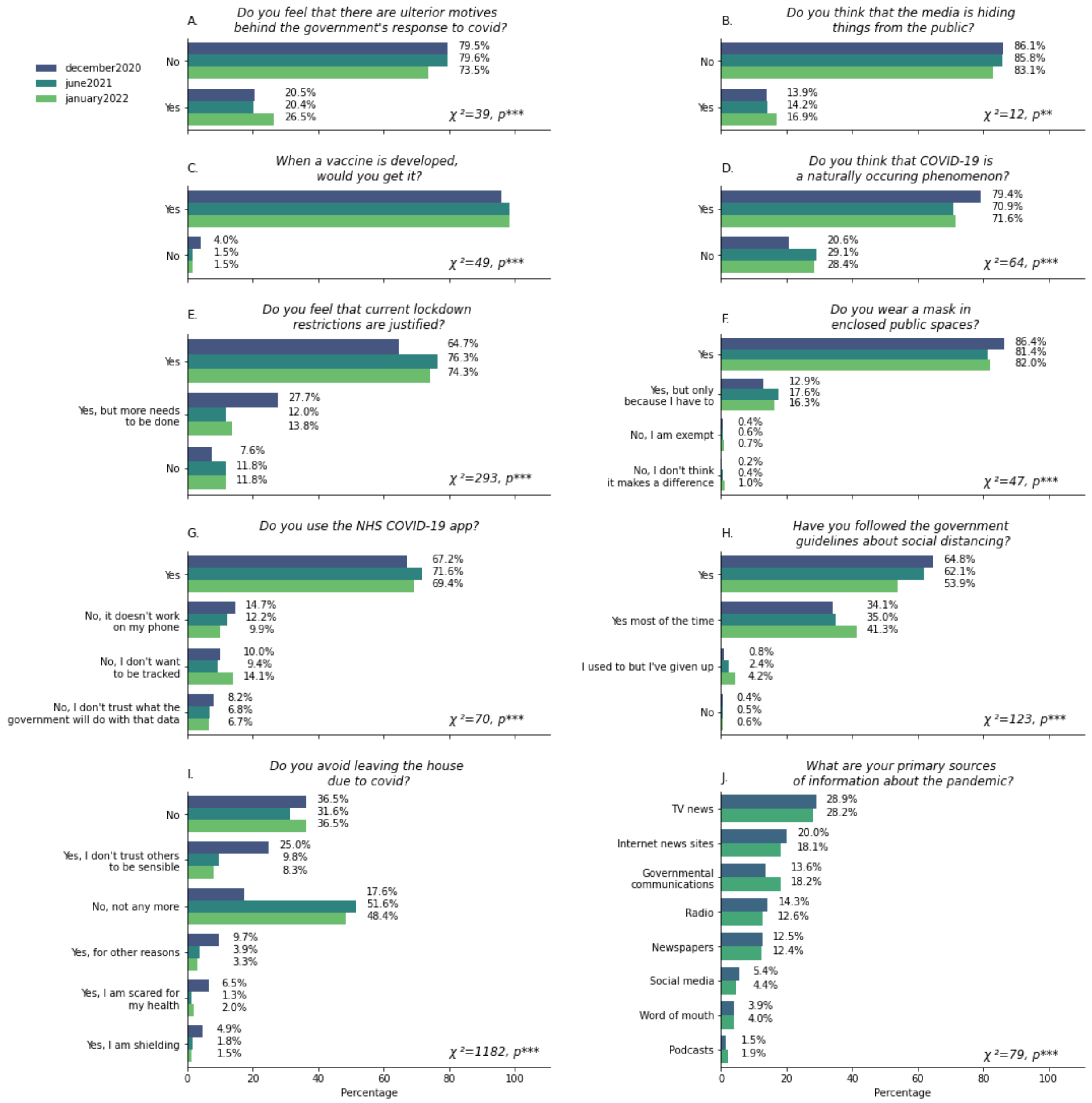

Longitudinal distribution of beliefs, compliance and media sources. The data presented only refers to the N=2797 respondents **who completed all three timepoints**. Panels A-E illustrate the proportions of various responses to belief-class questions. Panels F-I illustrate responses received to questions regarding compliance to measures that attempt to reduce COVID-19 transmission. Figure 1J illustrates the distribution of primary sources about the pandemic that people use. All x-axes show percentages. Each panel contains χ^2 and p values of tests for dissimilarity between the December 2020, June 2021 and January 2022 data. Answers are displayed in cascade order based on the most prevalent answers given in December 2020. Information regarding sources of

information about the pandemic was not collected in January 2022. Significance is denoted as *, **, and *** for p<0.05, p<0.01, and p<0.001, respectively.

Fig. S2.

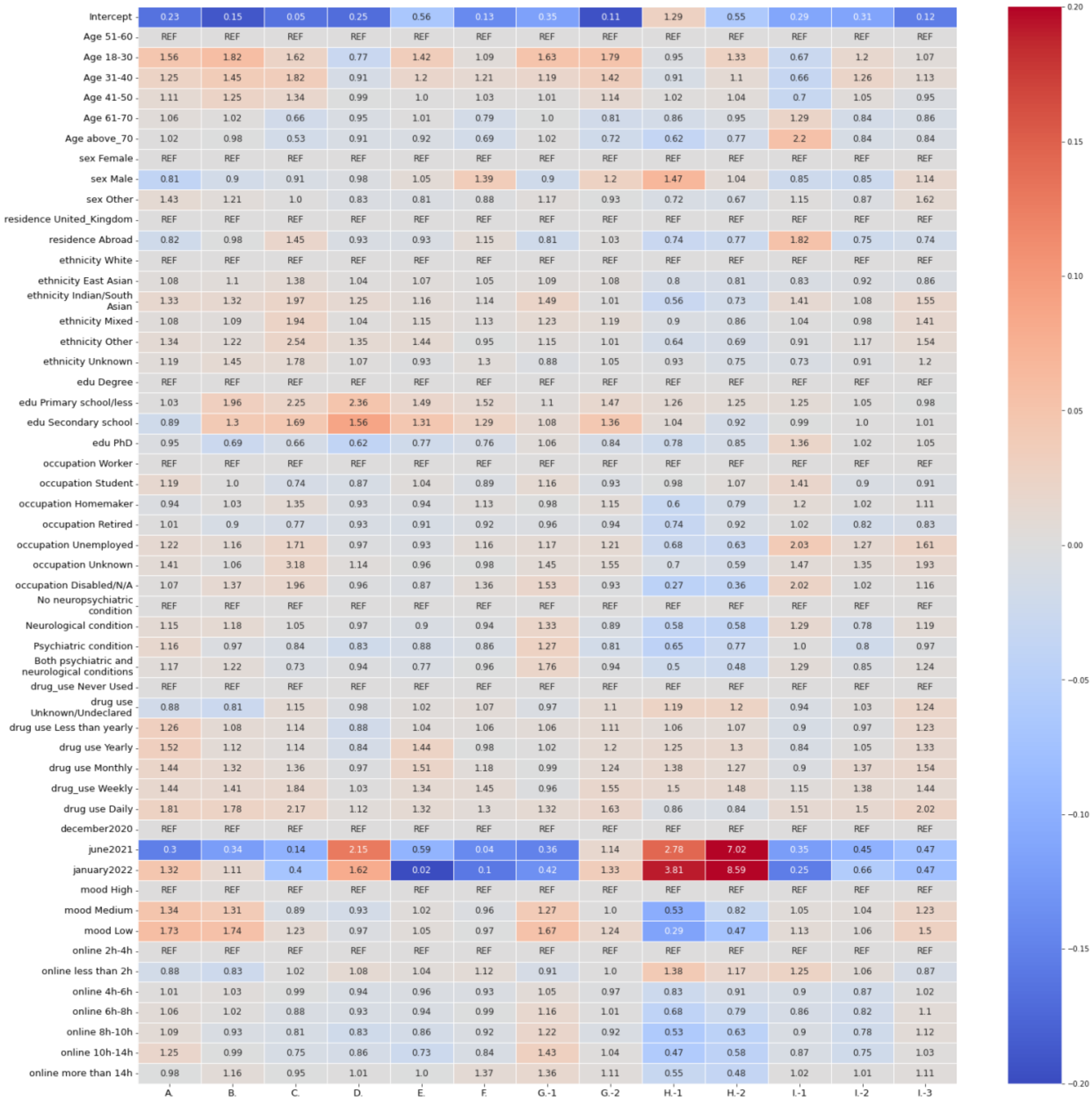

Figure S2. Odds and effect size of holding divergent opinions or being non-compliant associated with each predictor. The intensity represents Cohen's d effect size (d = 0.2 be considered a 'small' effect size, 0.5 represents a 'medium' effect size and 0.8 a 'large' effect size) and the annotations represent odds ratios relative to the 'REF' labelled predictors. A. -Assuming gov has ulterior motives,B.- Thinking the media is hiding things, C-Refuses vaccination, D.- Thinks the virus has

unnatural origins, E.- Seldom social distances', F.- Seldom wears a mask, G.- Considers restrictions unjustified, G.-Considers more restrictions are needed, H. - Does not avoid leaving the house, H.- Does not avoid leaving the house anymore, I.- The Track&Trace app does not work on their phone, I.- Does not want to be tracked, I. - Does not trust how the government will use Track&Trace data.

Fig. S3.

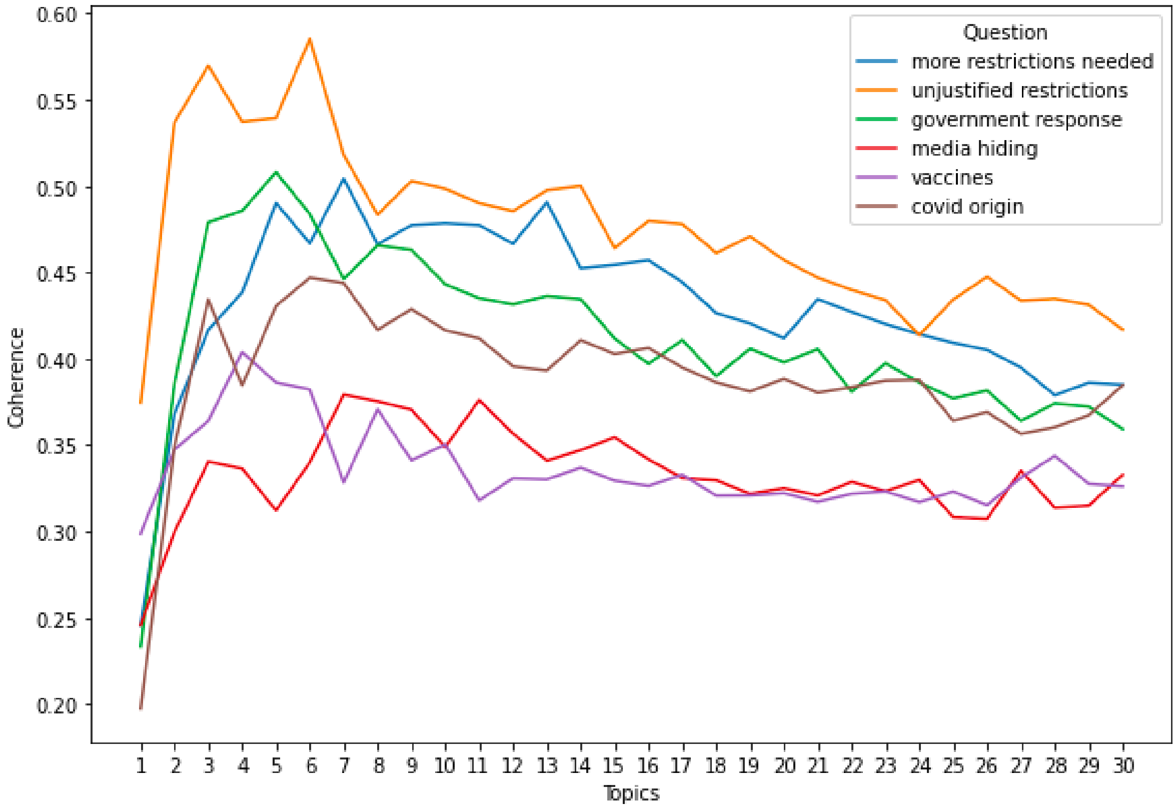

Topic modelling evaluation. In this figure we show the average coherence value for these questions per number of topics. Optimal topic numbers have been chosen based on the corresponding highest coherence value.

Fig. S4.

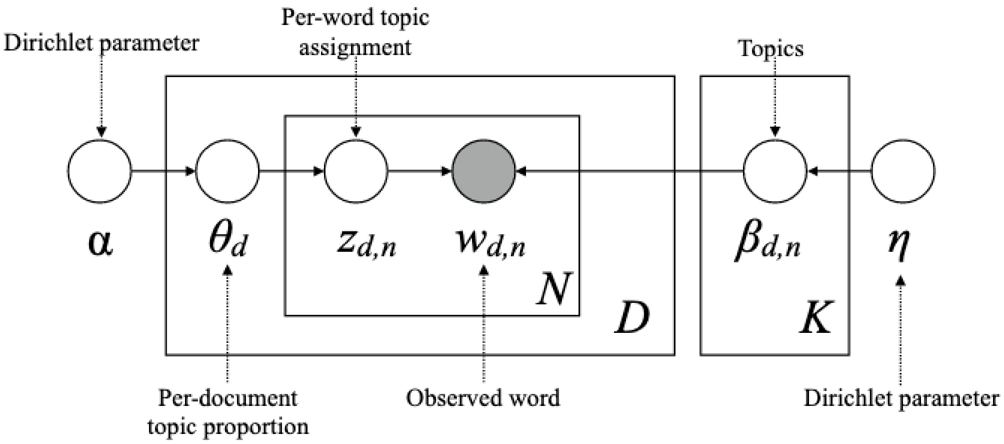

LDA represented as a graphical model. The nodes denote random variables. The edges represent the dependencies between random variables. The white nodes represent the hidden variables. The grey node represents the observed random variable. The boxes (plates) indicate replication. The figure has been adopted from Syed and Spruit 2017.

Fig. S5.

Question 1: Why do you think restrictions are not justified?						
Topic 1	Topic 2	Topic 3	Topic 4	Topic 5	Topic 6	Topic 7
Need	Case	People	Lockdown	Government	People	Virus
People	Rate	Rule	School	Late	Many	Spread
Virus	Still	Take	Need	Pandemic	Rule	Restriction
Health	Infection	Need	Close	Make	Mask	Control
Life	Rise	Follow	Work	Seem	Still	Country

Question 2: Why do you think more should be done?					
Topic 1	Topic 2	Topic 3	Topic 4	Topic 5	Topic 6
Health	Government	Lockdown	People	People	Vulnerable
Lockdown	Make	Tier	School	Life	Protect
Mental	Rule	Work	Open	COVID-19	People
Impact	Don't	Area	Close	Economy	Risk
Damage	Think	Case	Restriction	Dear	Need

Question 3: Why do you think the government has ulterior motives?				
Topic 1	Topic 2	Topic 3	Topic 4	Topic 5
Want	Government	Economy	Brexit	Contract
Don't	Decision	Health	Control	Money
Lockdown	Political	People	Government	Friend
People	Make	Try	Virus	Give
Government	Always	Life	COVID-19	Government

Question 4: Why do you think the media is hiding things?						
Topic 1	Topic 2	Topic 3	Topic 4	Topic 5	Topic 6	Topic 7
Don't	Make	Panic	Medium	Medium	Medium	COVID-19
Think	News	Never	Always	Information	News	People
Medium	Medium	Medium	Government	Truth	Bias	Death
Believe	Thing	People	Agenda	Government	Reporting	Number
Want	Self	Story	Control	Want	Fact	Case

Question 5: Why would you refuse COVID-19 vaccination?			
Topic 1	Topic 2	Topic 3	Topic 4
Vaccine	Don't	Risk	Effect
Test	Vaccine	Vaccine	Long
Rush	Know	Need	Term
Wait	Need	Virus	Enough
Sure	Take	COVID-19	Side

Question 6: What do you think the real origins of COVID-19 are?					
Topic 1	Topic 2	Topic 3	Topic 4	Topic 5	Topic 6
Health	Make	Animal	China	Chinese	Virus
Animal	Don't	Eat	Market	Laboratory	Experiment
Natural	Know	Market	Poor	Escape	Go
Virus	Think	People	Hygiene	Release	Think
Sure	Come	Human	Food	Virus	Wrong

Top 5 words per topic. Top 5 words are presented for each of the free text questions assessed.

Fig S6.

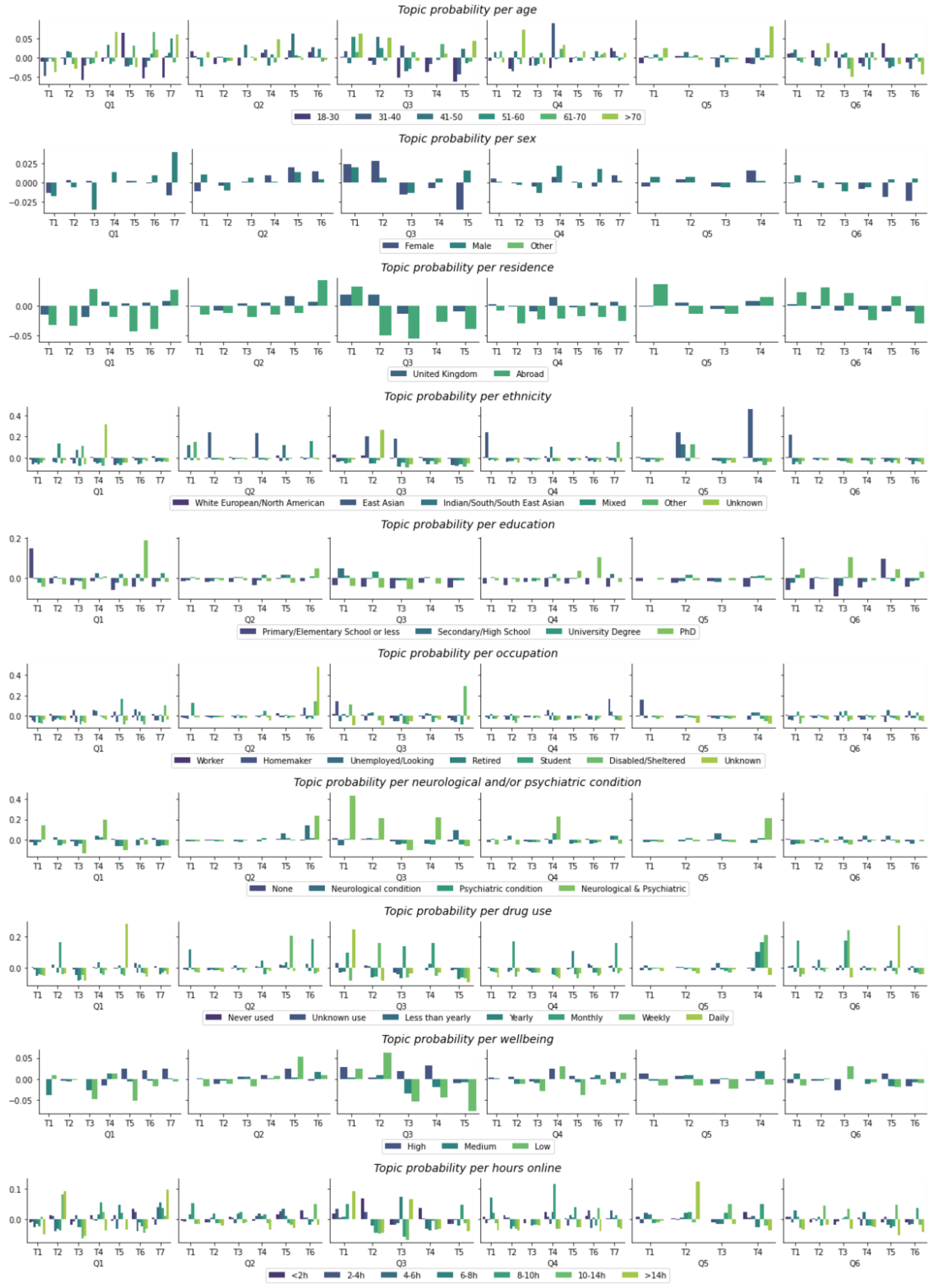

Probability changes of different topics in different sociodemographics from December 2020 to June 2021

Fig S7.

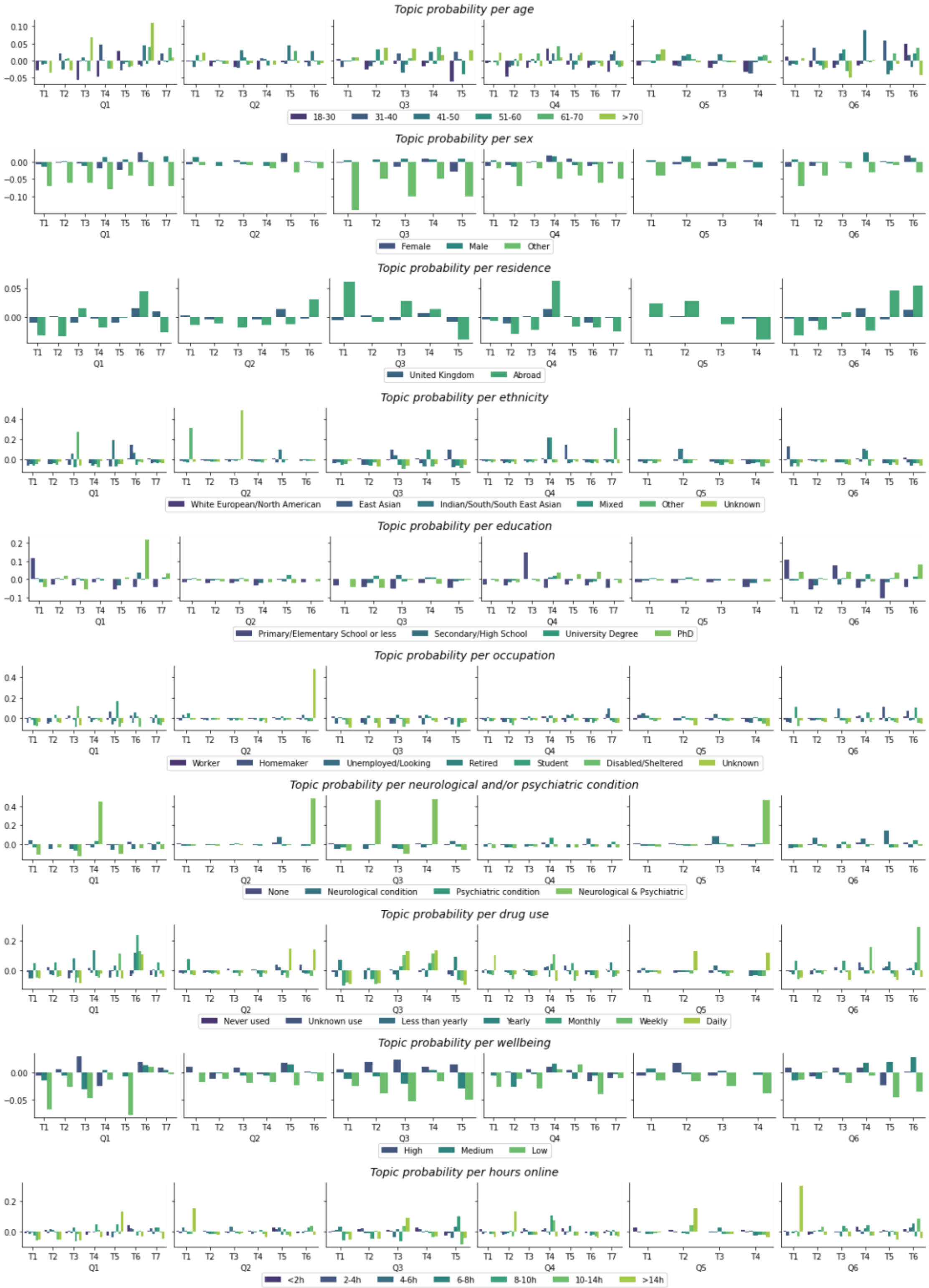
Topic probability per age
18-30
31-40
41-50
51-60
61-70
>70
Topic probability per sex
Female
Male
Other
Topic probability per residence
United Kingdom
Abroad
Topic probability per ethnicity
White European/North American
East Asian
Indian/South/South East Asian
Mixed
Other
Unknown
Topic probability per education
Primary/Elementary School or less
Secondary/High School
University Degree
PhD
Topic probability per occupation
Worker
Homemaker
Unemployed/Looking
Retired
Student
Disabled/Sheltered
Unknown
Topic probability per neurological and/or psychiatric condition
None
Neurological condition
Psychiatric condition
Neurological & Psychiatric
Topic probability per drug use
Never used
Unknown use
Less than yearly
Yearly
Monthly
Weekly
Daily
Topic probability per wellbeing
High
Medium
Low
Topic probability per hours online
<2h
2-4h
4-6h
6-8h
8-10h
10-14h
>14h

Probability changes of different topics in different sociodemographics from December 2020 to January 2022

Table S1.

Factor	Value	% Dec 2020	N Dec 2020	% June 2021	N June 2021	N Jan 2022	% Jan 2022	χ^2	p-value
**Age**	51 to 60	24.0	4965	24.4	3101	25.3	3541	86.03	***
	61 to 70	21.3	4422	22.3	2825	21.8	3056		
	41 to 50	17.2	3555	17.5	2224	18.7	2627		
	18 to 30	14.7	3053	13.7	1739	12.5	1754		
	31 to 40	14.7	3048	14.5	1845	15.3	2144		
	Above 70	8.1	1677	7.6	960	6.4	899		
**Sex**	Female	50.9	10645	52.2	6682	53.1	7484	18.62	***
	Male	48.6	10172	47.3	6051	46.5	6548		
	Other	0.5	105	0.5	63	0.4	58		
**Residence**	United Kingdom	93.4	19551	96.2	12305	95.8	13493	153.67	***

	Abroad	6.6	1371	3.8	491	4.2	597		
**Ethnicity**	White European or North American	90.7	18978	92.3	11807	91.7	12914	37.22	***
	Indian, South Asian or South-East Asian	2.1	443	1.7	216	1.8	253		
	Mixed ethnicity	2.1	443	1.9	240	1.9	266		
	East Asian	1.9	407	1.7	213	2.1	296		
	American Hispanic	1.7	358	1.4	178	1.4	204		
	Other	0.8	160	0.5	70	0.6	88		
	Unknown	0.6	133	0.6	72	0.5	69		
**Education**	University degree	61.4	12836	61.5	7869	62.4	8791	8.40	0.21
	Secondary school/High school diploma	32.1	6721	32.3	4132	31.1	4376		

	PhD	5.0	1040	4.8	620	5.1	718		
	Primary/Elementary school or less	1.6	325	1.4	175	1.5	205		
**Occupation**	Worker	60.4	12629	60.5	7737	62.8	8854	111.90	***
	Retired	26.1	5463	27.6	3533	25.8	3642		
	Student	6.3	1324	5.4	693	4.5	637		
	Unemployed/ Looking for work	3.3	682	2.7	343	2.7	375		
	Homemaker	2.4	502	2.6	331	2.9	409		
	Disabled/Not applicable/Sheltered employment	1.1	225	1.0	131	1.0	140		
	Unknown	0.5	97	0.2	28	0.2	33		

**Prescence of a neuropsychiatric or neurological condition**	None	87.7	18247	86.8	11062	86.4	12149	44.98	***
	I have a psychiatric condition	8.8	1823	9.0	1143	8.5	1199		
	I have a neurological condition	2.9	614	3.5	443	4.1	578		
	I have both a psychiatric and a neurological condition	0.6	133	0.7	93	0.9	129		
**Drug use**	Never used	52.9	11061	24.1	3088	24.1	3088	7058.92	***
	Unknown use	29.0	6062	49.3	6313	49.3	6313		
	Less than yearly	10.0	2101	19.6	2504	19.6	2504		
	Yearly	4.0	843	3.1	402	3.1	402		
	Monthly	1.8	386	1.9	238	1.9	238		
	Weekly	1.1	240	1.0	131	1.0	131		

	Daily	1.1	229	0.9	120	0.9	120		
**Level of wellbeing**	High wellbeing	58.8	12193	35.4	4527	34.7	4889	2681.64	***
	Medium wellbeing	34.2	7089	52.9	6773	54.5	7671		
	Low wellbeing	7.0	1448	11.7	1493	10.8	1528		
**Time per day online**	Between 2h and 4h	32.4	6689	31.0	3953	32.0	4508	117.03	***
	Less than 2h	21.5	4448	24.2	3088	22.5	3163		
	Between 4h and 6h	19.6	4048	17.1	2179	18.0	2538		
	Between 6h and 8h	11.9	2465	11.5	1468	11.0	1544		
	Between 8h and 10h	7.9	1626	9.6	1222	9.5	1331		
	Between 10h and 14h	4.8	987	5.1	654	5.4	757		

More than 14h	1.9	400	1.5	191	1.7	236

Participant Demographics. Demographics of participants surveyed at the different timepoints are presented alongside chi-squared statistics (χ^2). Significance stars for χ^2 analysis with * indicating $p<0.05$, ** indicating $p<0.01$, and *** indicating $p<0.001$.

Table S6.

	Divergence from popular opinion	Non-Compliance
	(1)	(2)
Intercept	0.01	-0.18***
	(0.02)	(0.02)
Age 18-30	0.27***	0.19***
	(0.02)	(0.02)
Age 31-40	0.11***	0.12***
	(0.02)	(0.02)
Age 41-50	0.06***	0.04***
	(0.01)	(0.01)
Age 61-70	-0.02*	-0.15***
	(0.01)	(0.01)
Age above 70	-0.05**	-0.30***
	(0.02)	(0.02)
Sex Male	-0.05***	0.12***
	(0.01)	(0.01)
Sex Other	0.06	-0.07
	(0.07)	(0.07)
Residence Abroad	-0.05**	-0.05**
	(0.02)	(0.02)
Ethnicity East Asian	0.07**	-0.01
	(0.03)	(0.03)
Ethnicity Indian/South Asian	0.19***	-0.04
	(0.03)	(0.03)
Ethnicity Mixed	0.07**	0.08**
	(0.03)	(0.03)
Ethnicity Other	0.12**	0.09
	(0.06)	(0.06)
Ethnicity Unknown	0.11*	0.06
	(0.06)	(0.06)

Education PhD	-0.12***	-0.12***
	(0.02)	(0.02)
Education Primary/Less	0.34***	0.09**
	(0.04)	(0.04)
Education Secondary/HighSchool	0.14***	0.04***
	(0.01)	(0.01)
Occupation Homemaker	-0.02	-0.04
	(0.04)	(0.04)
Occupation Retired	0.00	0.01
	(0.01)	(0.01)
Occupation Student	-0.03	-0.01
	(0.03)	(0.03)
Occupation Unemployed	0.03	0.02
	(0.04)	(0.04)
Occupation Unknown	0.40***	0.04
	(0.13)	(0.13)
Occupation Disabled or N/A	0.08	-0.32***
	(0.06)	(0.06)
Neurological condition present	0.09***	-0.19***
	(0.03)	(0.03)
Psychiatric condition present	0.03**	-0.14***
	(0.02)	(0.02)
Both neurological&psychiatric conditions present	0.18***	-0.32***
	(0.05)	(0.05)
Substance use Unknown	-0.02**	0.08***
	(0.01)	(0.01)
Substance use Less than yearly	0.05***	0.10***
	(0.01)	(0.01)
Substance use Yearly	0.07***	0.20***
	(0.03)	(0.03)
Substance use Monthly	0.13***	0.31***
	(0.03)	(0.03)
Substance use Weekly	0.18***	0.33***
	(0.04)	(0.04)
Substance use Daily	0.33***	0.24***
	(0.04)	(0.04)
Medium mood	-0.13***	0.13***
	(0.01)	(0.01)

Low mood	-0.20***	0.22***
	(0.01)	(0.01)
Time online <2h	-0.02*	0.07***
	(0.01)	(0.01)
Time online 4-6h	-0.00	-0.05***
	(0.01)	(0.01)
Time online 6-8h	0.02	-0.11***
	(0.02)	(0.02)
Time online 8-10h	0.00	-0.18***
	(0.02)	(0.02)
Time online 10-14h	0.08***	-0.24***
	(0.02)	(0.02)
Time online >14h	0.07**	-0.12***
	(0.04)	(0.04)
Observations	46,954	46,954
R2	0.04	0.04
Adjusted R2	0.04	0.04
Residual Std. Error	0.98 (df=46914)	0.98 (df=46914)
F Statistic	45.27*** (df=39; 46914)	52.53*** (df=39; 46914)
Note:	\n *p<0.1;\n **p<0.05;\n ***p<0.01\n	

Regression outputs of predicting scepticism and non-compliance from sociodemographic characteristics.

Table S2. (separate file)

Probabilities of answering questions about beliefs, compliance and media sources based on sociodemographic factors at the different timepoints.

TableS5. (separate file)

Chi-squared statistics per topic covary with sociodemographic factors.
